# METTL14‐mediated upregulation of lncRNA HOTAIR represses PP1α expression by promoting H3K4me1 demethylation in oxycodone‐treated mice

**DOI:** 10.1111/cns.14830

**Published:** 2024-07-24

**Authors:** Tian‐Cong Liu, Hong‐Xi Li, Yu‐Xiao Wan, Guang Shi, Yun‐Peng Zhao, Yi‐Fei Liu, Xin‐Yu Fan

**Affiliations:** ^1^ Department of Otolaryngology Shengjing Hospital of China Medical University Shenyang China; ^2^ Department of Pain Management Shengjing Hospital of China Medical University Shenyang China; ^3^ Department of Anesthesiology Shengjing Hospital of China Medical University Shenyang China; ^4^ Department of Neurology The People's Hospital of Liaoning Province Shenyang China; ^5^ Department of Pharmacy Shengjing Hospital of China Medical University Shenyang China

**Keywords:** H3K4me1, LSD1, m6A, METTL14, oxycodone

## Abstract

N6‐methyladenosine (m6A) methylation is a vital epigenetic mechanism associated with drug addiction. However, the relationship between m6A modification and oxycodone rewarding is less well explored. Based on an open field test, the present study evaluated oxycodone rewarding using chromatin immunoprecipitation PCR, immunofluorescence, and RNA sequencing. A marked increase in METTL14 protein and a decrease in PP1α protein due to oxycodone abundance in the striatal neurons were observed in a dose‐ and time‐dependent manner. Oxycodone markedly increased LSD1 expression, and decreased H3K4me1 expression in the striatum. In the open field test, intra‐striatal injection of METTL14 siRNA, HOTAIR siRNA, or LSD1 shRNA blocked oxycodone‐induced increase in locomotor activity. The downregulation of PP1α was also inhibited after treatment with METTL14/HOTAIR siRNA and LSD1 shRNA. Enhanced binding of LSD1 with CoRest and of CoRest with the PP1α gene induced by oxycodone was also reversed by LSD1 shRNA. In addition, H3K4me1 demethylation was also blocked by the treatment. In summary, the investigation confirmed that METTL14‐mediated upregulation of HOTAIR resulted in the repression of PP1α, which in turn facilitated the recruitment of LSD1, thus catalyzing H3K4me1 demethylation and promoting oxycodone addiction.

## Graphical abstract

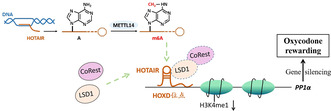

## 1 INTRODUCTION

Opioid abuse, a psychiatric disorder, has become a potential public health issue. The role of dopaminergic, mesocorticolimbic brain circuitry in the processing and perception of opioid addiction is well‐known.^1^, ^2^ While the literature targeting the mechanisms underlying dopamine reward circuitry is plentiful,^3^, ^4^, ^5^ much less is known about the relation between m6A methylation and opioid addiction. Several studies have demonstrated the association of N6‐methyladenosine (m6A) with the misuse of cocaine, nicotine, and alcohol.^6^, ^7^, ^8^

m6A, whose abundance in the mammalian transcriptome has been confirmed, is known to participate in many processes of brain function and neuronal development, including axon regeneration, cerebellar development, cortical neurogenesis, hippocampus‐dependent learning, and memory.^9^, ^10^, ^11^ In eukaryotic cells, m6A demethylation is catalyzed by demethylases ALKBH5 and FTO, while its methylation is catalyzed by a methyltransferase complex consisting of methyltransferase‐like 3 (METTL3) and 14 (METTL14).^12^ According to a previous study, aberrant chemical modifications of synaptic mRNAs have a role in the pathogenesis of neuropsychiatric disorders.^13^ In adult rodents, deficiency of FTO or METTL14 was reported to intervene with neurogenesis, memory, and dopaminergic signaling.^14^, ^15^ Although there exists some evidence relating to m6A methylation and drug addiction, the role of m6A in oxycodone rewarding is still an open question.

Histone methylation is closely related to transcriptional regulation and is involved in processes that induce drug abuse. Previous studies have shown that chronic cocaine decreases global levels of histone 3 lysine 9 tri‐methylation (H3K9me3) in the NAc.^16^ Repeated morphine downregulates NAc H3K9 di‐methylation (H3K9me2).^17^ H3K4 demethylation is regulated by the lysine‐specific demethylase (KDM) families LSD1 and KDM5, which demethylate H3K4me2/me1 to H3K4me0 and H3K4me3/me2 to H3K4me1, respectively.^18^ However, little is known about how oxycodone affects the levels of H3K4 methylation.

In order to uncover the potential epitranscriptomic role of m6A methylation in oxycodone abuse, three different doses of oxycodone were administered to a group of mice for 9 days. Subsequently, after 4 days of oxycodone withdrawal, striatum samples from the experimental mice were collected for RNA‐sequencing (RNA‐seq) to detect m6A methylation‐associated enzymes and then examine m6A‐related epigenetic alterations. An upregulation in the levels of METTL14, lncRNA HOX transcript antisense RNA (lncRNA HOTAIR), and lysine demethylase LSD1 was detected in all oxycodone‐treated mice. As confirmed by RNA‐seq analyses, treatment with siRNA METTL14/HOTAIR or shRNA LSD1 alleviated oxycodone‐induced increases in locomotor activity, throwing some light on the potential uses of this new approach in opioid deaddiction.

## 2 MATERIALS AND METHODS

### 2.1 Animals

**FIGURE 1:**
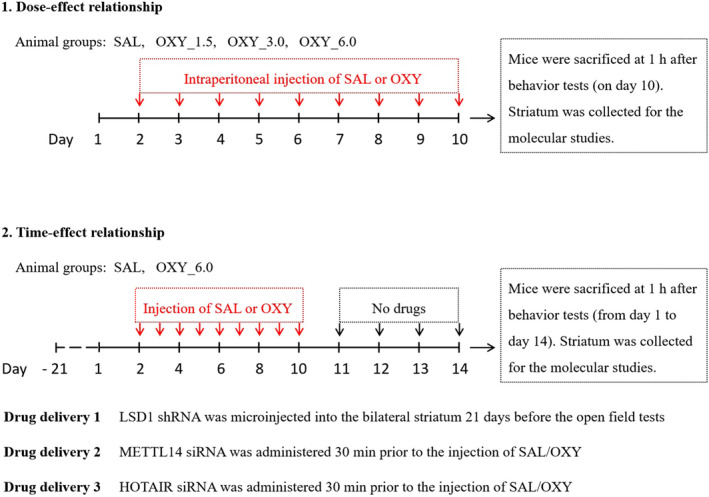
The experimental design of the present study.

Animal use protocol (2021PS221K) was approved by the Laboratory Animal Care Committee of Shengjing Hospital of China Medical University. For this study, 310 male C57BL/6J mice (18–22 g) were purchased from Beijing HFK Bioscience Co., Ltd. (China). All the mice were housed in an SPF room with a temperature of 23 ± 1°C, humidity maintained at 60 ± 15%, and a 12:12 h light/dark cycle, with access to sufficient food and water. For anesthetic purposes, 5.0% isoflurane for induction and 2.5% isoflurane for maintenance were used. The experimental design of the present study was shown in Figure 1.

### 2.2 Drug delivery

Three siRNAs targeting METTL14 mRNA or HOTAIR as well as a scrambled nontargeting oligo (scRNA; 5′‐TUCUCUTGCTUGUCAUACUTT‐3′) were obtained from GenePharma (China). Their transfection efficiency in the primary neurons was assessed. Adeno‐associated virus (AAV2) expressing LSD1 shRNA (AAV‐LSD1 shRNA‐EGFP, 4 μL of 10^12^ vg/mL, 200 nL/min) and a negative control (AAV‐LSD1 ncRNA‐EGFP) were purchased from HANBIO (China). siRNA or shRNA was administered to the mice by intra‐striatal injection. Catheterization was performed as follows: two 26‐gauge stainless steel guide cannulas were inserted into the striatum bilaterally (AP +0.8 mm, ML ±1.6 mm, DV +3.0 mm) and stabilized on the skull using dental cement. Two dummy cannulas with the same dimensions as guide cannulas were inserted from the top to prevent clogging and infection. After allowing recovery for a week, a 31‐gauge injector tube was inserted into the guide cannula, and siRNA or scRNA (1.0 μL per mouse) was delivered 30 min prior to open field tests. In our preliminary experiment, we observed that shRNA required at least 3 weeks to exhibit its functional effects. Conversely, the inhibition effects of siRNA on the expression of HOTAIR were notably faster. Therefore, the administration paradigm for shRNA and siRNA differs significantly in this study. A previous study on the functional characteristics of siRNA and shRNA has also confirmed the difference in their action times.^19^ Prior to this, LSD1 shRNA or ncRNA was microinjected into the bilateral striatum (2.0 μL per mouse) 3 weeks before the open field tests. Saline or oxycodone (1.5, 3.0, and 6.0 mg/kg) was injected intraperitoneally (i.p., 0.1 mL/10 g) from day 2 to day 10.

### 2.3 Open field test

To assess locomotor activity, each mouse from different groups (*n* = 8 animals in each group) was placed in a square arena (50 cm in length, width, and height) under dim light. Mice were dropped in the corner every day and allowed to move freely in the arena. The arena was cleaned thoroughly using 75% alcohol solution between each trial to remove odor cues. The movement of every individual mouse was recorded for 15 min and analyzed the travel distance of each mouse using video‐tracking software (EthoVision XT, Noldus, the Netherlands).

### 2.4 Western blotting

During striatum collection, all the mice (*n* = 4 animals in each group) were perfused with 50 mL phosphate‐buffered saline (PBS). Protein extraction was carried out by sonication with RIPA buffer for 3 min on ice, followed by mearing its concentration using a BCA kit (Cat# PC0020; Solarbio, China). Thirty micrograms of the extracted protein were separated with 10% SDS–PAGE and transferred onto a PVDF membrane (Cat# 88518; ThermoFisher, USA). The membrane was blocked with 5% nonfat milk for 1 h followed by overnight incubation with corresponding primary antibodies. It was then washed with TBST for 15 min and then incubated with HRP‐conjugated anti‐rabbit lgG secondary antibodies (Cat# S0001; Affinity, USA) at room temperature for 1 h. Protein bands were measured with ECL and quantified using ImageJ software (version 2). The primary antibodies used in the study include METTL14 (Cat# Ab308576; Abcam, UK), PP1α (Cat# BM4356; Boster, China), CoRest (Cat# Ab183711; Abcam), H3K4me3 (Cat# Ab213224; Abcam), H3K4me2 (Cat# Ab32356; Abcam), H3K4me1 (Cat# Ab176877; Abcam), LSD1 (Cat# BM4356; Boster), KDM5A (Cat# Ab194286; Abcam), β‐actin (Cat# AF7018; Affinity, USA), and H3 (Cat# BM4389; Boster).

### 2.5 Immunofluorescence

After harvesting and perfusion with 4% paraformaldehyde (Cat# P0099; Beyotime, China), the whole brains (*n* = 4 animals in each group) were fixed in 4% paraformaldehyde for 48 h, dehydrated with 10/20/30% sucrose for 72 h, and then thinly (15 μm) sliced. These thin slices were permeabilized in TBS with Triton X‐100 for 10 min and incubated with a peroxidase‐blocking buffer for 10 min at room temperature. Subsequently, after washing with TBS three times, blocking using 5% goat serum (Cat# C0265; Beyotime), and incubation overnight with primary antibodies against PP1α (Cat# BM4356; Boster), METTL14 (Cat# Ab308576; Abcam, UK), and NeuN (Cat# Ab104224) at 4°C, these slices were incubated with secondary antibodies (DyLight 488 or 594 Conjugated AffiniPure Goat, Anti‐rabbit, or Mouse lgG(H + L); Cat# BA1126, BA1127, BA1141, BA1142; Boster, China) for 1 h at room temperature. Images obtained using Nikon Ci confocal microscopy (Japan) with excitation wavelengths 405, 488, and 561 nm were analyzed with ImageJ software (NIH).

### 2.6 Real‐time quantitative polymerase chain reaction (RT–qPCR)

Total RNA in the striatum (*n* = 4 animals in each group) was extracted using TRIzol reagent (Cat# 12183555; ThermoFisher, USA) and reverse‐transcribed into cDNA using an EasyScript Kit (Cat# AE341‐02; TransGen Biotech, China). Real‐time PCR (Stratagene Mx3000P; Agilent Technologies, Germany) was performed in triplicate on a 20 μL sample (0.4 μL ROX reference dye, 10 μM primers, 10 ng DNA, 10 μL 2× SYBR Green qPCR Master Mix). The PCR was performed as follows: (1) initial denaturation at 95°C for 30 s; (2) 35 cycles: denaturation at 95°C for 15 s followed by annealing and extension step at 60°C for 1 min. The primers for PP1α, METTL14, HOTAIR, and glyceraldehyde 3‐phosphate dehydrogenase (GADPH) were as follows: PP1α: forward, 5′‐CGGCTGTTTGAGTATGGTGG‐3′, reverse, 5′‐GCAGTCAGTGAACGTCTTCC‐3′; METTL14: forward, 5′‐TTCTGGGGAAGGATTGGACC‐3′, reverse, 5′‐ACGGTTCCTTTGATCCCCAT‐3′; HOTAIR: forward, 5′‐GGACCGACGCCTTCCTTATA‐3′, reverse, 5′‐TGCGTGTCTTCTGTCCTTCT‐3′; GADPH: forward, 5′‐GGGTCCCAGCTTAGGTTCAT‐3′, reverse, 5′‐CATTCTCGGCCTTGACTGTG‐3′. The comparative method (2^−ΔΔCt^) was used to determine the relative expression of PP1α, METTL14, and HOTAIR.

### 2.7 Co‐immunoprecipitation (Co‐IP)

After collection, the striatum (*n* = 4 animals in each group) was lysed using a lysis buffer (Cat# P0013; Beyotime, China), and the lysates were incubated with anti‐CoREST antibody for 8 h followed by the addition of A/G Plus‐Agarose (Cat# sc‐2003; Santa Cruz, USA). The protein‐antibody‐bead mixtures were incubated at 4°C overnight with rotary agitation. All mixtures were washed using a lysis buffer followed by the addition of 5× SDS loading buffer (Cat# P0015; Beyotime, China). Western blotting was used to detect the expression of KDM1A and CoREST. An IB assay for CoREST was used as a control.

### 2.8 Chromatin immunoprecipitation‐PCR (ChIP‐PCR)

For the ChIP‐PCR trial (*n* = 4 animals in each group), a ChIP assay kit was used (Cat# 17–295; Millipore, Germany) as per the manufacturer's instructions. Striatum homogenization was initiated with 1% formaldehyde for 15 min and terminated by adding 125 mM glycine. The pellets so obtained after centrifugation were lysed using an SDS lysis buffer with a protease inhibitor cocktail. Under suitable sonication conditions, 600–800 bp DNA fragments were obtained. These were recycled and subjected to immunoprecipitation with 10 μg anti‐CoREST or control IgG overnight; 20% of the sample was used as input. The protein‐DNA precipitant underwent PCR amplification for PP1α promoter fragments. Experiments were performed in triplicate using the following primers: P1: foward, 5′‐TAAGGAGCCCAGATTAGCGG‐3′, 5′‐reverse, TCTTCTACAACCTGGGCCAG‐3′; P1: forward, 5′‐TAAGGAGCCCAGATTAGCGG‐3′, 5′‐reverse, TCTTCTACAACCTGGGCCAG‐3′; P2: forward, 5′‐CCCCAATGATGAGCCCTGTA‐3′, reverse, 5′‐GTATCTTCTCTTGCCTGCGC‐3′; P3: forward, 5′‐CTGGCCCAGGTTGTAGAAGA‐3′, reverse, 5′‐GCCCTGGGAGATTAGATGCT‐3′; P4: forward, 5′‐CCCCAATGATGAGCCCTGTA‐3′, reverse, 5′‐GTATCTTCTCTTGCCTGCGC‐3′; P5: forward, 5′‐AAGCTAGCTGGGAAGGGATC‐3′, reverse, 5′‐CCACCATACTCAAACAGCCG‐3′; P6: forward, 5′‐AGAAGCTCAACCTGGACTCC‐3′, reverse, 5′‐GGATTTGAGGCACAGACCAC‐3′.

### 2.9 RNA‐sequencing (RNA‐seq)

Total RNA was extracted from the striatum (*n* = 3 animals in each group), and the quantification of lncRNA, ncRNA, and mRNA followed. For the construction of sequencing libraries, the MGISEQ‐2000RS High‐Throughput Sequencing Reagent Kit (Cat#1000012554; MGI, China) was used. For quality checking, the Standard Sensitivity RNA Analysis Kit (DNF‐471‐0500) and the Fragment Analyzer (Agilent 5300) were employed. SOAPnuke (v1.5.6) was used for filtering the sequencing data. Quality‐checked libraries were sequenced on the DNBseq platform using MGISEQ‐2000 with 100PE sequencing. Library construction, lncRNA‐seq, ncRNA‐seq, and RNA‐seq, as well as data collection and mapping were outsourced to HuaDa Gene Biotechnology (Shenzhen, China).

### 2.10 Quantification of RNA m6A

The EpiQuik m6A RNA Methylation Quantification Kit (Cat# P9005‐48; Epigenetek, USA) was used to quantify overall m6A methylation as per manufacturer's instructions. Briefly, 200 ng RNA (*n* = 4 animals in each group) was transferred to 96 wells followed by the addition of antibody solution. The level of m6A absorbance in each well at a wavelength of 450 nm was evaluated. All computations were performed based on the standard curve.

### 2.11 Statistical analysis

Data were expressed as mean ± standard error of the mean (SEM). All analyses were carried out using SPSS 13.0 software (SPSS Inc., USA). The Shapiro–Wilk test was used to test the normality of data variance, and data homogeneity was assessed with Levene's test. When analyzing the results of molecular studies, between‐group differences were tested using one‐way analysis of variance (ANOVA) with Tukey's post hoc test for multiple comparisons or using Student's unpaired *t*‐test. Results of open field tests were analyzed using two‐way ANOVA with repeated measurements followed by Tukey's post hoc test for differences over time. Individual Tukey's post hoc test between groups was only run when the F‐value was *p* < 0.05. The level of statistical significance was set at *p* < 0.05.

## 3 RESULTS

### 3.1 Oxycodone altered mRNAs, ncRNAs, and lncRNAs levels in the striatum

**FIGURE 2:**
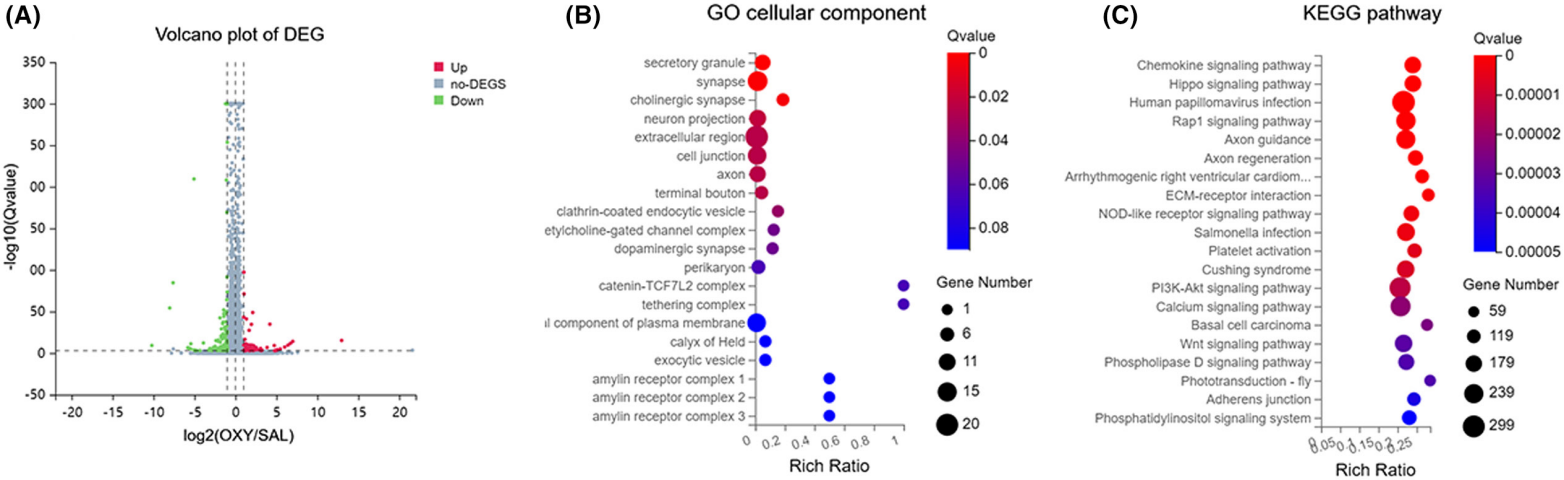
Oxycodone altered the expression of mRNAs, ncRNAs, and lncRNAs in the striatum. (A) Volcanic plot of differentially expressed genes. The X coordinate is log_2_ (fold change) and the Y coordinate is log_10_ (*Q*‐value). Each point stands for a gene. Red points represent significantly upregulated genes. Green points represent significantly downregulated genes. Gray points represent genes with nonsignificant expression. (B) Representation of the 20 cellular components with the most significant differential gene enrichment. The X coordinate presents the rich ratio and the Y coordinate presents the cellular component terms. (C) Bubble plot of KEGG enrichment analysis. The X coordinate is the enrichment ratio of differentially expressed genes and the Y coordinate is the pathway.

RNA‐seq was used to investigate the pathology of oxycodone abuse. According to our results, 15 mRNAs, 8 ncRNAs, and 39 lncRNAs were markedly downregulated, while 91 mRNAs, 20 ncRNAs, and 50 lncRNAs were upregulated after oxycodone injection (Figure 2A). The details of differential gene expression were displayed in Table S1. The Gene Ontology (GO) study suggested the enrichment of the most number of differentially expressed genes in the extracellular region, notably those in secretory granule, synapse, and cholinergic synapse. The highest rich ratio of GO enrichment was in the catenin‐TCF7L2 complex and tethering complex (Figure 2B). The BGI online platform (www.bgi.com) was used to analyze the KEGG (Kyoto Encyclopedia of Genes and Genomes) pathways of different genes. Accordingly, differentially expressed genes were enriched in the chemokine signaling pathway and the hippo signaling pathway. The highest number of differential genes was enriched for the human papillomavirus infection (Figure 2C). It was noted that the expression of METTL14 and PP1α in the striatum was affected by oxycodone injection. Thus, we explored the involvement of m6A methylation in the regulation of PP1α expression under conditions of oxycodone abuse.

### 3.2 Oxycodone downregulated striatal PP1α underlying m6A methylation

**FIGURE 3:**
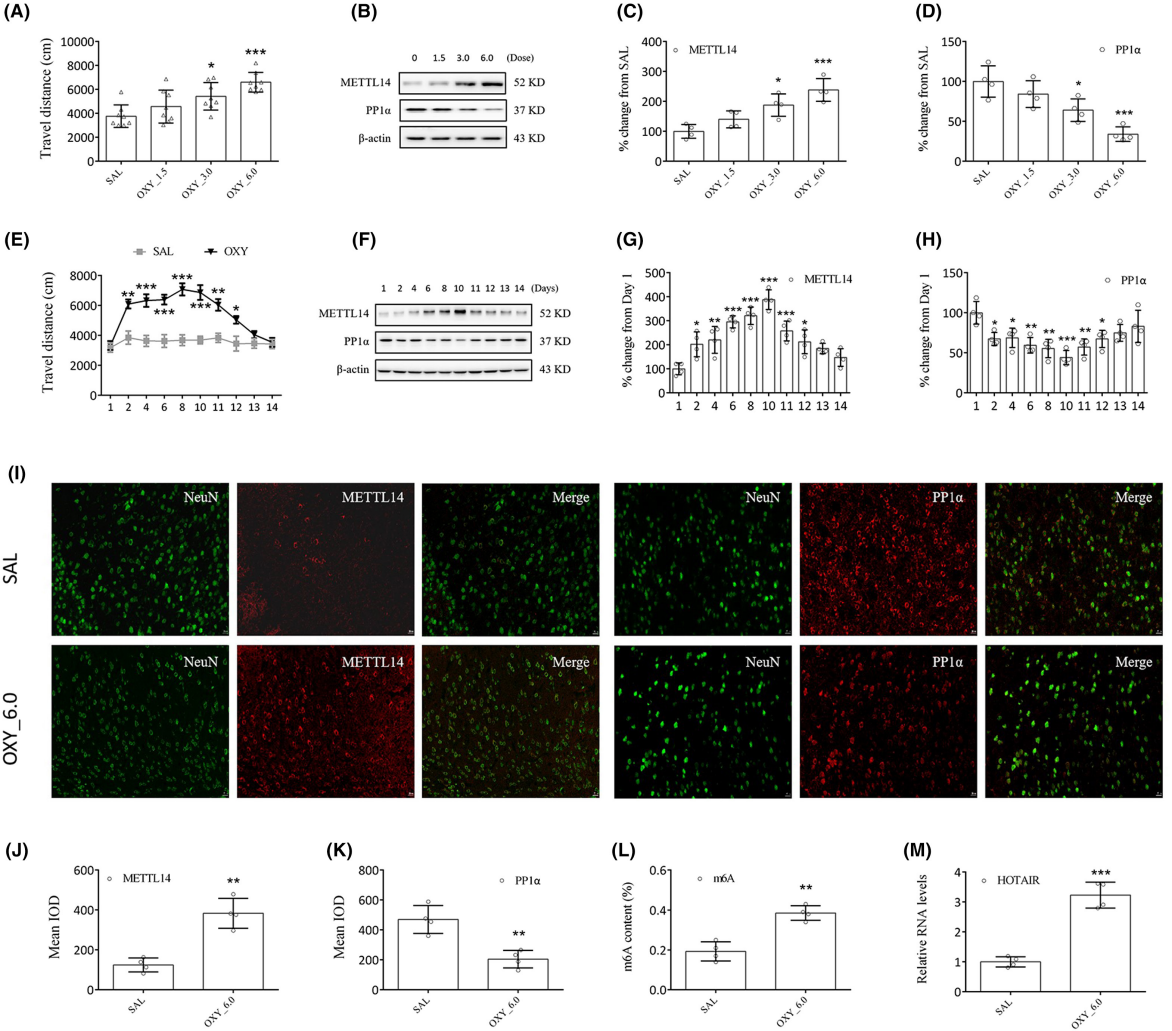
Oxycodone downregulated striatal PP1α underlying m6A methylation. (A) Oxycodone (1.5, 3.0, and 6.0 mg/kg) was injected to all test animals. (B–D) Western blotting shows a significant increase in METTL14 expression and a decrease in PP1α expression. (E) Oxycodone markedly increased the movement of mice. *p < 0.05, **p < 0.01, ***p < 0.001, vs. baseline (day 1), two‐way ANOVA. (F–H) Western blotting shows the expressions of METTL14 and PP1α in striatal neurons. *p < 0.05, **p < 0.01, ***p < 0.001, vs. SAL group or day 1, one‐way ANOVA. Representative images of double immunofluorescence staining (I) of METTL14 or PP1α (red) and NeuN (green). METTL14 (J), m6A (L), and HOTAIR (M) levels were upregulated, while PP1α level (K) was downregulated in oxycodone‐treated mice. Scale bar (E) = 20 μm. *p < 0.05, **p < 0.01, ***p < 0.001, vs. SAL group or day 1, one‐way ANOVA.

Oxycodone at dosages of 1.5, 3.0, and 6.0 mg/kg was administered to all experimental mice. Compared to the SAL group, the movement of the OXY group was marked elevated (OXY_3.0: *p* < 0.05; OXY_6.0: *p* < 0.001, Figure 3A). Thus, it was decided to use oxycodone at a dosage of 6.0 mg/kg in subsequent experiments. The results of Western blotting and RT–qPCR show a significant increase in METTL14 expression and a significant decrease in PP1α expression for OXY compared to SAL group (Western blotting: OXY_3.0: *p* < 0.05; OXY_6.0: *p* < 0.001, Figure 3B–D; RT‐qPCR: OXY_3.0: *p* < 0.01; OXY_6.0: *p* < 0.005, Figure S1A,B).

Daily changes in METTL14 and PP1α levels were evaluated as well. Oxycodone administration (6.0 mg/kg, days 2 to 10) markedly elevated the movement of mice (vs. SAL, day 2, 11: *p* < 0.01; day 4, 6, 8, 10: *p* < 0.001; day 12: *p* < 0.05, Figure 3E). From day 11, all the mice were subjected to oxycodone withdrawal. As can be expected, the results of Western blotting (Figure 3F–H) and RT–qPCR (Figure S1C,D) show a significant increase in METTL14 expression and a significant decrease in PP1α expression in all oxycodone‐treated mice. Compared to the SAL group (day 1), the OXY group demonstrated significant differences in METTL14 and PP1α expressions from day 2, reaching a peak on day 10.

We noted the colocalization of METTL14 and PP1α with neuronal nuclear protein (NeuN, a marker of neuron, Figure 3I) but not with glial fibrillary acidic protein (GFAP, a marker of satellite glial cell, Figure S1E). Increased METTL14 (*p* < 0.01, Figure 3J), m6A (*p* < 0.01, Figure 3L), and HOTAIR (*p* < 0.001, Figure 3M) expressions and reduced PP1α expression (*p* < 0.01, Figure 3K) in the striatum were characteristics of oxycodone treatment compared to the SAL group.

### 3.3 Blockade of METTL14 or HOTAIR decreased locomotor activity in oxycodone‐treated mice

**FIGURE 4:**
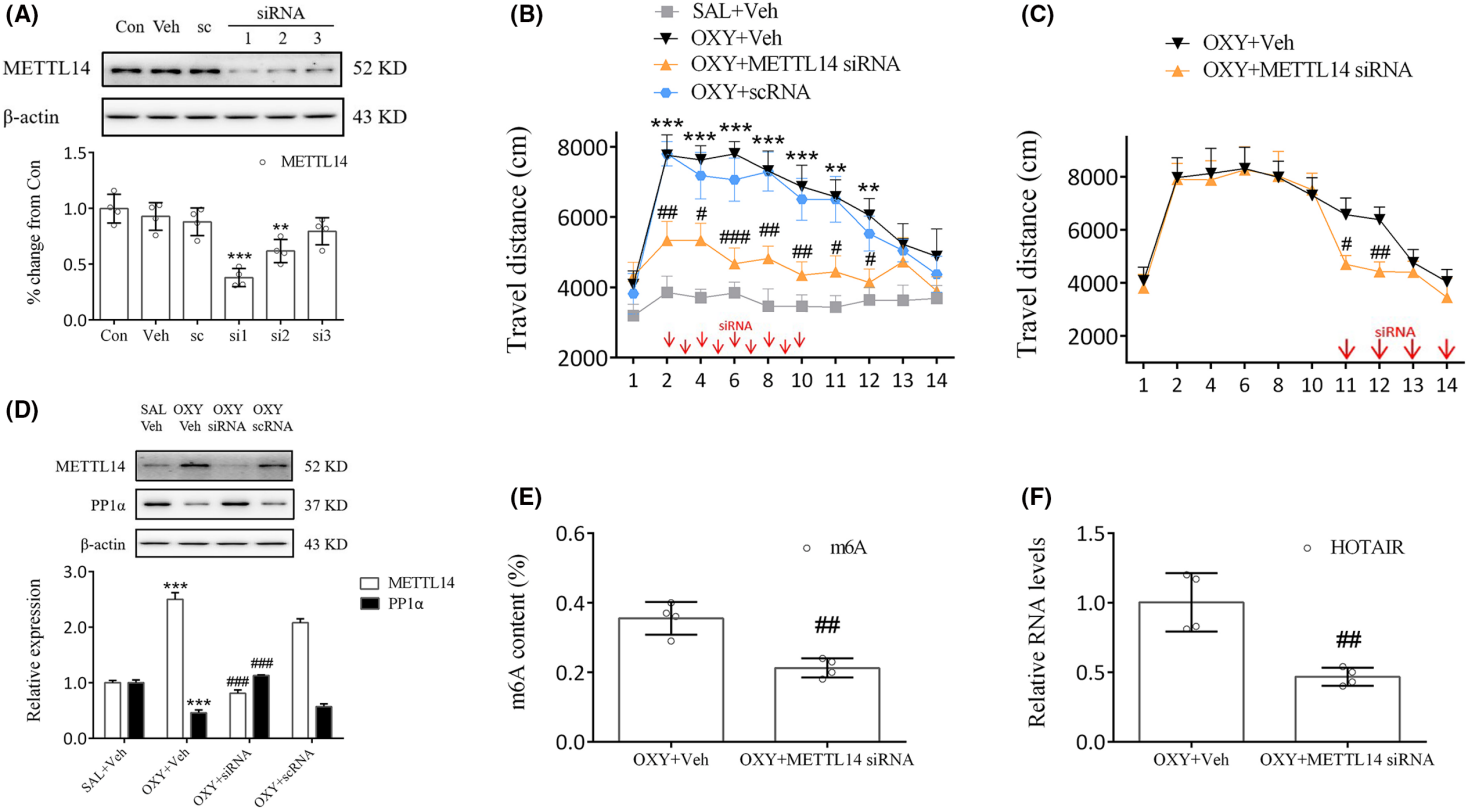
Blockade of METTL14 lowered locomotor activity in oxycodone‐treated mice. (A) Expression of METTL14 in the primary cells was inhibited by METTL14 siRNA treatment. METTL14 siRNA1 (si1) was chosen because of its high silencing efficiency. **p < 0.01, ***p < 0.001, vs. scrambled nontargeting oligo (sc group), one‐way ANOVA. (B) Injection of METTL14 siRNA significantly decreased the movement of mice. **p < 0.01, ***p < 0.001, vs. SAL+Veh group; ^#^ *p* < 0.05, ^##^ *p* < 0.01, ^###^ *p* < 0.001, vs. OXY + Veh group, two‐way ANOVA. (C) Significantly lesser movement was observed on days 11–12. METTL14 siRNA treatment markedly increased the expression of PP1α (D) while decreasing m6A (E) and HOTAIR levels (F). ***p < 0.001, vs. SAL+Veh group; ^##^ *p* < 0.01, ^###^ *p* < 0.001, vs. OXY + Veh group, one‐way ANOVA.

Identifying the role of METTL14 in oxycodone abuse is the next course of our work. To that end, METTL14 siRNAs were prepared and investigated, whose results indicated that siRNA1 (si1) significantly inhibited METTL14 expression (*p* < 0.001, Figure 4A). Therefore, further experimentation continued with si1. Behavioral study showed that treatment of mice with METTL14 siRNA (days 2–10) 30 min prior to oxycodone injection significantly reduced their movement compared to the OXY + Veh group (days 2, 8, 10: *p* < 0.01; days 4, 11, 12: *p* < 0.05; days 6: *p* < 0.001, Figure 4B). Even during the phase of oxycodone withdrawal, METTL14 siRNA administration continued on days 11 to 14, during which time a marked decrease in movement was noted on days 11 and 12 (day 11: *p* < 0.05; day 12: *p* < 0.01, Figure 4C). Meanwhile, as our study results suggest, METTL14 siRNA significantly increased PP1α expression (*p* < 0.001, Figure 4D) but decreased METTL14 (*p* < 0.001, Figure 4D), m6A (*p* < 0.01, Figure 4E), and HOTAIR expressions (*p* < 0.01, Figure 4F) in oxycodone‐treated mice. Therefore, the blockade of METTL14 in the striatum could inhibit oxycodone abuse underlying m6A methylation.

**FIGURE 5:**
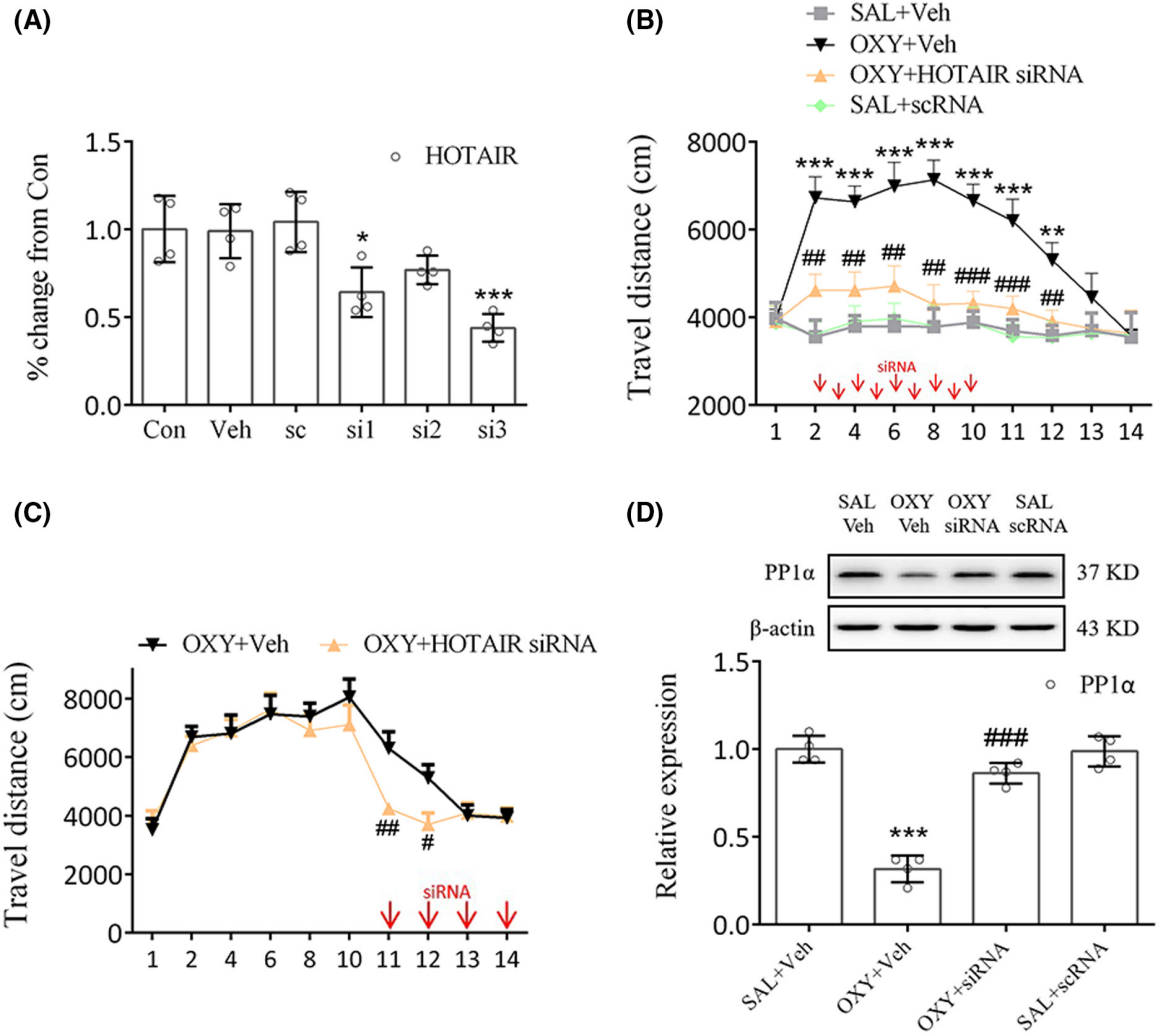
Blockade of HOTAIR lowered locomotor activity in oxycodone‐treated mice. (A) Expression of HOTAIR in the primary cells was inhibited by HOTAIR siRNA treatment. HOTAIR siRNA3 (si3) was chosen because of its high‐silencing efficiency. *p < 0.05, ***p < 0.001, vs. sc group, one‐way ANOVA. (B) Injection of HOTAIR siRNA significantly lowered the movement of oxycodone‐treated mice. **p < 0.01, ***p < 0.001, vs. SAL+Veh group; ^##^ *p* < 0.01, ^###^ *p* < 0.001, vs. OXY + Veh group, two‐way ANOVA. (C) Significantly lesser movement was observed on days 11–12. (D) HOTAIR siRNA treatment markedly increased the expression of PP1α in oxycodone‐treated mice. ***p < 0.001, vs. SAL+Veh group; ^###^ *p* < 0.001, vs. OXY + Veh group, one‐way ANOVA.

HOTAIR siRNA3 (si3) was chosen because it significantly reduced HOTAIR levels in the primary cells (*p* < 0.001, Figure 5A). Behavioral tests showed that HOTAIR siRNA injection 30 min prior to oxycodone administration markedly decreased movement compared to the OXY + Veh group on days 2–12 (days 2, 4, 6, 8, 12: *p* < 0.01; day 10, 11: *p* < 0.001, Figure 5B). Moreover, HOTAIR siRNA injection on days 11–14 markedly decreased movement compared to the OXY + Veh group on days 11–12 (day 11: *p* < 0.01; day 12: *p* < 0.05, Figure 5C). METTL14 or HOTAIR siRNA failed to hinder the oxycodone‐induced increase in travel distance observed on days 13–14 (Figures 4B,C, 5B,C), presumably due to the spontaneous extinction of the oxycodone‐treated mice's locomotor hyperactivity on the third day following the final drug administration (SAL+Veh vs. OXY + Veh, Figure 4B). Consistent with this finding, a previous study demonstrated that the locomotor sensitization triggered by oxycodone vanished on the second day after the last administration of oxycodone.^20^ Meanwhile, significant differences in PP1α expression (*p* < 0.001, Figure 5D) were noted between the OXY + HOTAIR siRNA group and the OXY + Veh group. From these results, the involvement of HOTAIR in regulating PP1α expression in the striatum can be inferred.

### 3.4 LSD1‐mediated demethylation of H3K4me1 inhibited PP1α expression in oxycodone‐treated mice

**FIGURE 6:**
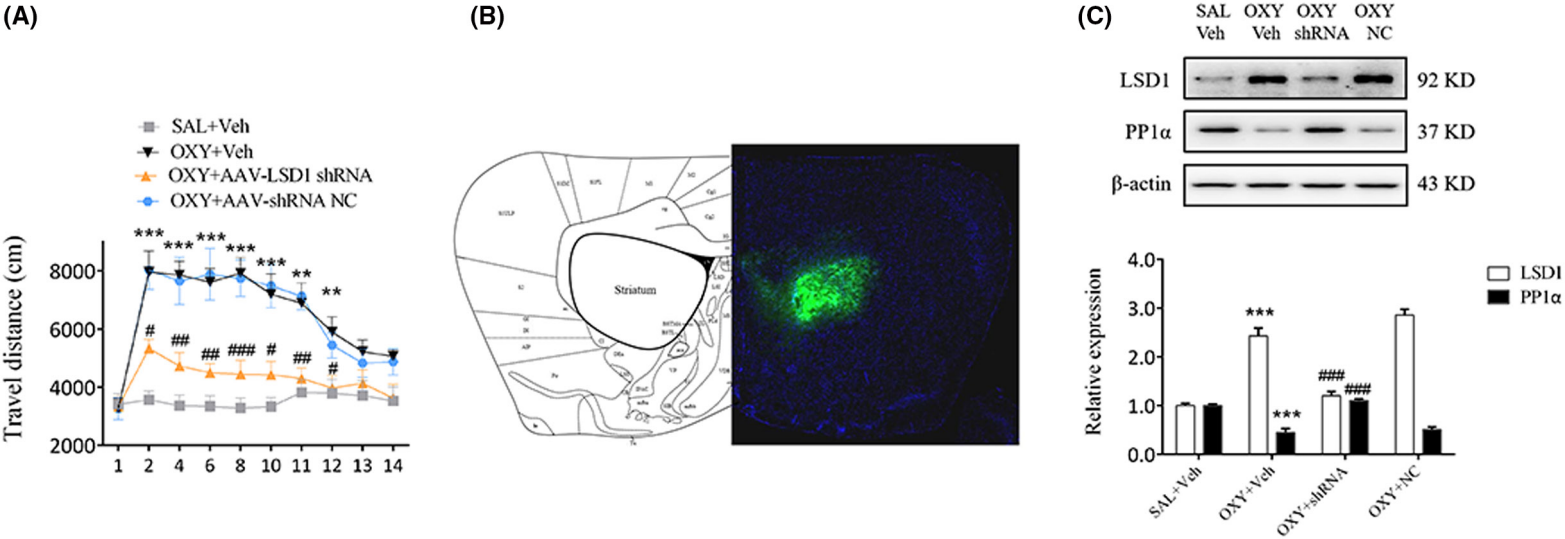
Treatment with AVV‐LSD1 shRNA inhibited locomotor activity in oxycodone‐treated mice. (A) Injecting AVV‐LSD1 shRNA 3 weeks prior to the open field test markedly decreased the locomotor activity of mice. **p < 0.01, ***p < 0.001, vs. SAL+Veh group; ^#^ *p* < 0.05, ^##^ *p* < 0.01, ^###^ *p* < 0.001, vs. OXY + Veh group, two‐way ANOVA. (B) Fluorescence localization of AVV in the striatum. (C) Western blot showing increased expression of PP1α after LSD1 shRNA administration in oxycodone‐treated mice. ***p < 0.001, vs. SAL+Veh group; ^###^ *p* < 0.001, vs. OXY + Veh group, one‐way ANOVA.

AVV‐LSD1 shRNA was administered bilaterally into mice striatum 3 weeks prior to the open field tests. As we noted, treatment of mice with AVV‐LSD1 shRNA, but not with AVV‐shRNA NC, markedly reduced their movement from day 2 to day 12 (day 2, 10, 12: *p* < 0.05; day 4, 6, 11: *p* < 0.01; day 8: *p* < 0.001, Figure 6A), indicating that LSD1 inhibition in the striatum could block oxycodone‐induced increase in locomotor activity. On day 14 the distribution of AVV in each mouse was assessed via full‐scan fluorescence an hour after open field tests (Figure 6B). According to its findings, the blockade of LSD1 using shRNAs elevated PP1α expression in oxycodone‐treated mice (*p* < 0.001, Figure 6C), suggesting that LSD1 may be involved in the regulation of PP1α protein.

**FIGURE 7:**
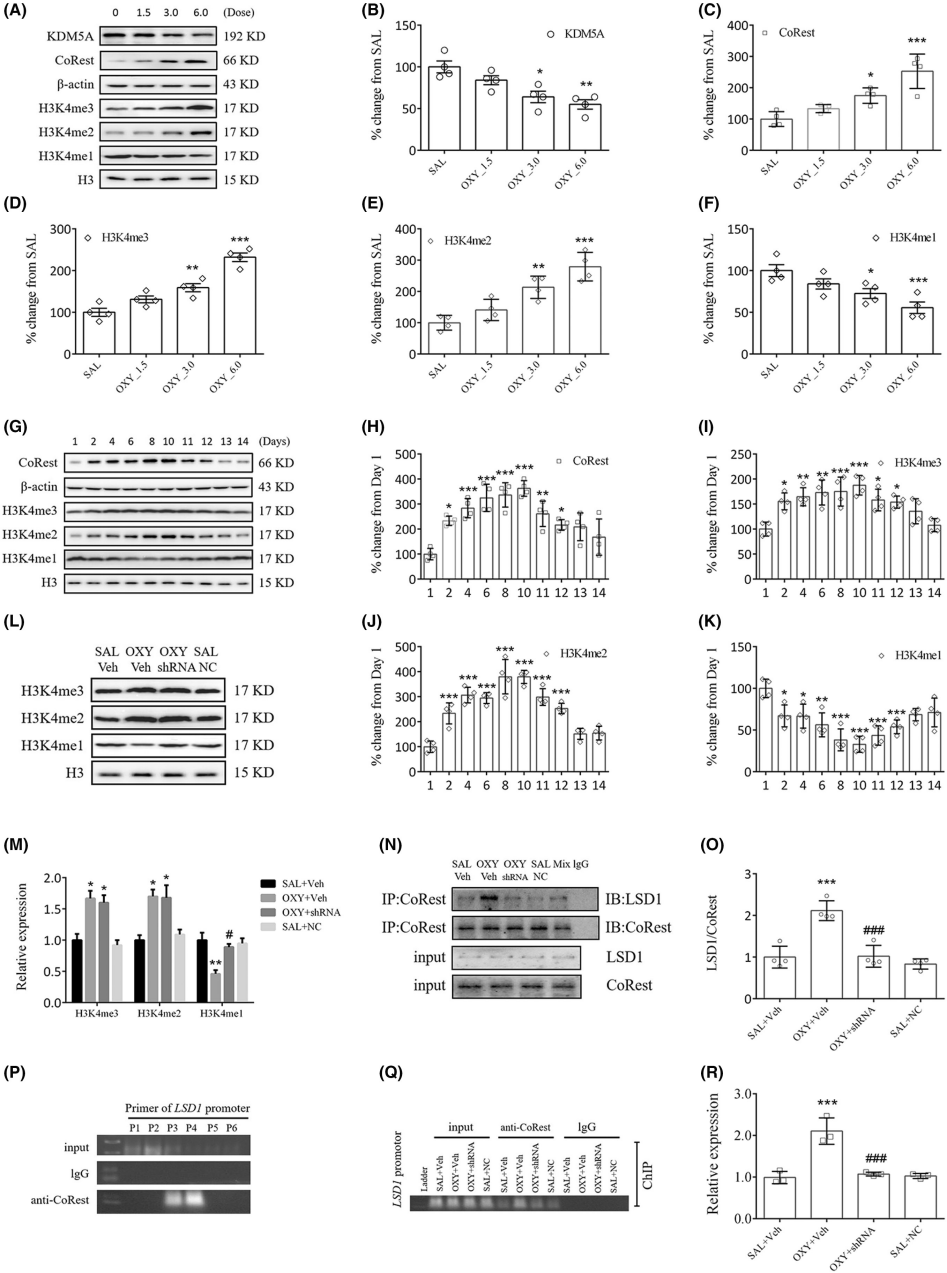
LSD1‐mediated demethylation of H3K4me1 inhibited PP1α expression in oxycodone‐treated mice. (A–F) Dose–effect relationship between expressions of KDM5A, CoRest, H3K4me3/me2/me1, and dosage of oxycodone. (G–K) Time–effect relationship between expressions of CoRest, H3K4me3/me2/me1, and time at which oxycodone was administered. *p < 0.05, **p < 0.01, ***p < 0.001, vs. SAL+Veh group, one‐way ANOVA. LSD1 shRNA administration significantly increased H3K4me1 expression (L, M), lowered binding of LSD1 and CoRest (N, O) as well as binding of CoRest with the PP1α gene (P–R) in oxycodone‐treated mice. ***p < 0.001, vs. SAL+Veh group; ^###^ *p* < 0.001, vs. OXY + Veh group, one‐way ANOVA.

As we know, KDM5A induces demethylation of H3K4me3/me2, and LSD1 demethylates H3K4me2/me1. At different doses in this study, oxycodone markedly reduced KDM5A and H3K4me1 expression (KDM5A: OXY_3.0, *p* < 0.05, OXY_6.0, *p* < 0.01; H3K4me1: OXY_3.0, *p* < 0.05, OXY_6.0, *p* < 0.001), while elevated CoRest, H3K4me3 and H3K4me2 expressions (CoRest: OXY_3.0, *p* < 0.05, OXY_6.0, *p* < 0.001; H3K4me3: OXY_3.0, *p* < 0.01, OXY_6.0, *p* < 0.001; H3K4me2: OXY_3.0, *p* < 0.01, OXY_6.0, *p* < 0.001, Figure 7A–F). Based on immunofluorescent studies, oxycodone markedly elevated CoRest expression in striatal neurons (Figure S1F). The significant increases in CoRest, H3K4me3 and H3K4me2 expressions, and decrease in H3K4me1 expression that began on day 2 reached a peak on day 10 and persisted up to day 12 (Figure 7G–K). Treatment with LSD1 shRNA prevented H3K4me1 level from reducing in oxycodone‐treated mice (*p* < 0.05, Figure 7L,M). Meanwhile, treatment with LSD1 shRNA reversed oxycodone‐induced binding of LSD1 and CoRest (*p* < 0.001, Figure 7N,O).

The role of LSD1 in the binding of CoRest with the PP1α gene was investigated with ChIP–PCR. For the detection of the PP1α gene, six primers (P1–P6) were designed, in which the offspring was only observed in P3 and P4 when linking the input DNA fragment to the anti‐CoRest antibody (Figure 7P). PP1α gene fragments of similar intensity were observed for all the input DNAs. Of note, oxycodone significantly increased PP1α gene fragments in the OXY + Veh group compared to the SAL+Veh group (*p* < 0.001, Figure 7Q,R). However, treatment with LSD1 shRNA blocked this increase (*p* < 0.001, Figure 7Q,R). In sum, all our findings lead to a suggestion that oxycodone‐induced increases in LSD1 will facilitate the binding of CoRest with the PP1α gene via H3K4me1 demethylation.

## 4 DISCUSSION

This study on chronic oxycodone exposure has demonstrated that METTL14‐mediated lncRNA HOTAIR methylation promoted the interaction of LSD1 with the PP1α gene, and its resulting downregulation contributed to oxycodone addiction. The study also confirmed that the blockade of METTL14, HOTAIR, or LSD1 in the striatum inhibited locomotor activity in oxycodone‐treated mice.

Existing evidence shows the connections of m6A methylation and its regulatory proteins with learning and memory.^21^, ^22^ Although narcotics can lead to profound alterations in the expressions of lncRNAs, mRNAs, and ncRNAs underlying both histone and DNA methylation,^4^, ^23^ the potential role of m6A methylation in oxycodone addiction remains an open question. To the best of our knowledge, the present study is the first to report METTL14‐mediated m6A methylation in mouse striatum as a result of oxycodone addiction. As the present findings showed, oxycodone administration markedly elevated METTL14 expression and reduced PP1α expression in a time‐ and dose‐dependent manner. Subsequent to oxycodone treatment, the inhibition of METTL14 in the striatum could increase the expression of PP1α and decrease locomotor activity, suggesting that METTL14 is a regulator of reward motivation by altering the PP1α pathway, marked by m6A methylation. Similarly, studies have demonstrated the impact of FTO on the CREB pathway, confirming its involvement in the locomotor‐activating effects of cocaine.^15^, ^24^ The role of METTL14 in the development of major neuropsychiatric diseases such as Huntington's disease and Alzheimer's disease has been well researched.^25^, ^26^ Therefore, the mechanism of action of METTL14 in regulating the PP1α expression is worth investigating.

Our RNA‐seq analysis shows sufficient proof of elevation in the expression of lncRNA HOTAIR in the striatum due to oxycodone addiction. HOTAIR plays a coordinating role in gene expression and activating chromatin‐modifying enzymes. It plays a part in gene silencing by facilitating H3K4 demethylation and/or H3K27 methylation via directing LSD1 and/or PRC2 complex to the target genes.^27^ HOTAIR is also connected with epigenetic regulation, poor survival, and immune escape.^28^, ^29^ In a recent study, HOTAIR inhibition reduced the expression of a range of key dopamine neuron specification factors, proving the functional role of HOTAIR in dopamine neuron development and differentiation.^30^ In this study, treatment with HOTAIR siRNA significantly blocked both increases in locomotor activity and decreases in PP1α expression induced by oxycodone, suggesting that HOTAIR is a vital epigenetic regulator of the PP1α protein. In addition, treatment with LSD1 shRNA blocked oxycodone‐induced increases in locomotor activity and decreases in PP1α expression. Meanwhile, oxycodone administration significantly elevated the expression of CoRest and reduced the expression of H3K4me1 at the same time, suggesting that LSD1‐mediated H3K4me1 demethylation triggered some gene expression via chromatin remodeling. It was reported that H3K4me1 and H3K36me3 have the strongest association with splicing indicating they play a significant role in alternative splicing in brain reward tissue.^31^ In the study, oxycodone also reduced the expression of KDM5A, which induced the upregulation of H3K4me3/me2 level. In line with our results, a previous study found the opposite trend of LSD1 and KDM5A in patients with alopecia areata. It shows that alopecia areata increased KDM5A, MLL, SETD7, and G9A expression, as well as reduced LSD1, KDM4A, and KDM4B expression.^32^ We speculated that H3K4 methylation maintains an equilibrium state, where oxycodone induces demethylation of H3K4me1 and methylation of H3K4me3/me2. Although we can not make the conclusion on which enzyme (LSD1 or KDM5A) plays the leading role, our results showed the status of H3K4 methylation at this time point during oxycodone rewarding. The involvement of the LSD1/CoRest complex in the repression of gene transcription has been recently reported.^33^ From that perspective, a conclusion can be drawn from our results that oxycodone promoted the binding of CoRest with LSD1 in the striatum. However, these oxycodone‐mediated effects were blocked by LSD1 shRNA administration. Now the question that arises: Whether the LSD1/CoRest complex promoted H3K4me1 demethylation and, in turn, repressed PP1α expression?

According to one study, H3K4me2 demethylation at the *FosB* gene induced morphine‐conditioned place preference in rats under chronic stress.^34^ Similarly, another study suggests LSD1‐mediated demethylation of H3K9me2 in the amygdala also resulted in alcohol abuse in adolescents.^35^ We hypothesize that oxycodone‐induced upregulation of the LSD1–CoRest complex through H3K4me1 demethylation in the striatum may induce the binding of the complex with the PP1α gene, triggering the expression of PP1α protein. We ran the ChIP–PCR experiment to measure the binding of the LSD1–CoRest complex with the PP1α gene, which demonstrated that oxycodone administration led to a significant increase in the interaction of the LSD1‐CoRest complex with the PP1α gene. Such binding can be blocked by treatment with LSD1 shRNA, indicating that LSD1 is a potent source of defense against oxycodone‐induced interaction of the LSD1–CoRest complex with the PP1α gene.

## 5 CONCLUSIONS

The present study brings to light evidence suggesting that METTL14‐mediated upregulation of HOTAIR has a potency to repress the expression of PP1α and facilitate the recruitment of LSD1, thus catalyzing H3K4me1 demethylation and promoting oxycodone addiction.

## AUTHOR CONTRIBUTIONS

TCL, HXL, GS: Conception, methodology, and study design; YXW, YPZ, YFL: data collection; TCL, YXW: data analysis and manuscript drafting; XYF: manuscript revision. All the authors read and approved the final manuscript.

## FUNDING INFORMATION

This study was supported by the National Natural Science Foundation of China, No. 82101567; 345 Talent Project of Shengjing Hospital of China Medical University, No. M1312; China International Medical Foundation, No. Z‐2021‐46‐2101; Doctoral Research Foundation Project of Liaoning Province, No. 2022‐BS‐130.

## CONFLICT OF INTEREST STATEMENT

The authors declare that there is no conflict of interest.

## Supporting information


DataS1

FigureS1

TableS1

## Data Availability

Data will be made available on request from the authors.

## References

[1] Zhang Y , Liang Y , Randesi M , Yuferov V , Zhao C , Kreek MJ . Chronic oxycodone self‐administration altered reward‐related genes in the ventral and dorsal striatum of C57BL/6J mice: an RNA‐seq analysis. Neuroscience. 2018;393:333‐349. doi:10.1016/j.neuroscience.2018.07.032 30059705

[2] Koob GF , Volkow ND . Neurobiology of addiction: a neurocircuitry analysis. Lancet Psychiatry. 2016;3(8):760‐773. doi:10.1016/S2215-0366(16)00104-8 27475769 PMC6135092

[3] Emmanuel D , Lina KB . Opioid receptors: drivers to addiction? Nat Rev Neurosci. 2018;19:499‐514. doi:10.1038/s41583-018-0028-x 29934561

[4] Browne CJ , Godino A , Salery M , Nestler EJ . Epigenetic mechanisms of opioid addiction. Biol Psychiatry. 2020;87(1):22‐33. 31477236 10.1016/j.biopsych.2019.06.027 PMC6898774

[5] Jull D , Gondin AB , Zastrow MEV , et al. Opioid pharmacology under the microscope. Mol Pharmacol. 2020;98(4):425‐432. doi:10.1124/mol.119.119321 32198210 PMC7562971

[6] Xue A , Huang Y , Li M , Wei Q , Bu Q . Comprehensive analysis of differential m6A RNA Methylomes in the hippocampus of cocaine‐conditioned mice. Mol Neurobiol. 2021;58(8):3759‐3768. 33826069 10.1007/s12035-021-02363-4

[7] Liu B , Xia L , Li Y , et al. Prenatal nicotine exposure raises male blood pressure via FTO‐mediated NOX2/ROS signaling. Hypertension. 2023;81:240‐251. 37795601 10.1161/HYPERTENSIONAHA.123.21766 PMC10873091

[8] Liu Y , Koo JS , Zhang H . Chronic intermittent ethanol exposure‐induced m6A modifications around mRNA stop codons of opioid receptor genes. Epigenetics. 2024;19(1):2294515. 38118075 10.1080/15592294.2023.2294515 PMC10761033

[9] Wang CX , Cui GS , Liu X , et al. METTL3‐mediated m6A modification is required for cerebellar development. PLoS Biol. 2018;16(6):e2004880. 29879109 10.1371/journal.pbio.2004880 PMC6021109

[10] Weng YL , Wang X , An R , et al. Epitranscriptomic m6A regulation of axon regeneration in the adult mammalian nervous system. Neuron. 2018;97(2):313‐325.e6. 29346752 10.1016/j.neuron.2017.12.036 PMC5777326

[11] Shi H , Zhang X , Weng YL , et al. m6A facilitates hippocampus‐dependent learning and memory through YTHDF1. Nature. 2018;563(7730):249‐253. 30401835 10.1038/s41586-018-0666-1 PMC6226095

[12] Zhao BS , Roundtree IA , He C . Post‐transcriptional gene regulation by mRNA modifications. Nat Rev Mol Cell Biol. 2017;18(1):31‐42. 27808276 10.1038/nrm.2016.132 PMC5167638

[13] Merkurjev D , Hong WT , Iida K , et al. Synaptic N6‐methyladenosine (m6A) epitranscriptome reveals functional partitioning of localized transcripts. Nat Neurosci. 2018;21(7):1004‐1014. 29950670 10.1038/s41593-018-0173-6

[14] Li L , Zang L , Zhang F , et al. Fat mass and obesity‐associated (FTO) protein regulates adult neurogenesis. Hum Mol Genet. 2017;26(13):2398‐2411. 28398475 10.1093/hmg/ddx128 PMC6192412

[15] Hess ME , Hess S , Meyer KD , et al. The fat mass and obesity associated gene (Fto) regulates activity of the dopaminergic midbrain circuitry. Nat Neurosci. 2013;16(8):1042‐1048. 23817550 10.1038/nn.3449

[16] Maze I , Feng J , Wilkinson MB , Sun H , Shen L , Nestler EJ . Cocaine dynamically regulates heterochromatin and repetitive element unsilencing in nucleus accumbens. Proc Natl Acad Sci USA. 2011;108(7):3035‐3040. 21300862 10.1073/pnas.1015483108 PMC3041122

[17] Sun H , Maze I , Dietz DM , et al. Morphine epigenomically regulates behavior through alterations in histone H3 lysine 9 dimethylation in the nucleus accumbens. J Neurosci. 2012;32(48):17454‐17464. 23197736 10.1523/JNEUROSCI.1357-12.2012 PMC3516048

[18] Heward J , Koniali L , D'Avola A , et al. KDM5 inhibition offers a novel therapeutic strategy for the treatment of KMT2D mutant lymphomas. Blood. 2021;138(5):370‐381. 33786580 10.1182/blood.2020008743 PMC8351530

[19] Pushparaj PN , Aarthi JJ , Manikandan J , Kumar SD . siRNA, miRNA, and shRNA: in vivo applications. J Dent Res. 2008;87(11):992‐1003. 18946005 10.1177/154405910808701109

[20] Sierra S , Muchhala KH , Jessup DK , et al. Sex‐specific role for serotonin 5‐HT2A receptor in modulation of opioid‐induced antinociception and reward in mice. Neuropharmacology. 2022;209:108988. 35183539 10.1016/j.neuropharm.2022.108988 PMC8934299

[21] Zhang Z , Wang M , Xie D , et al. METTL3‐mediated N6‐methyladenosine mRNA modification enhances long‐term memory consolidation. Cell Res. 2018;28(11):1050‐1061. 30297870 10.1038/s41422-018-0092-9 PMC6218447

[22] Koranda JL , Dore L , Shi H , et al. Mettl14 is essential for Epitranscriptomic regulation of striatal function and learning. Neuron. 2018;99(2):283‐292.e5. 30056831 10.1016/j.neuron.2018.06.007 PMC6082022

[23] Vaher K , Anier K , Jürgenson M , Harro J , Kalda A . Cocaine‐induced changes in behaviour and DNA methylation in rats are influenced by inter‐individual differences in spontaneous exploratory activity. J Psychopharmacol. 2020;34(6):680‐692. 32338111 10.1177/0269881120916137

[24] Lin L , Hales CM , Garber K , Jin P . Fat mass and obesity‐associated (FTO) protein interacts with CaMKII and modulates the activity of CREB signaling pathway. Hum Mol Genet. 2014;23(12):3299‐3306. 24488767 10.1093/hmg/ddu043 PMC4030783

[25] Lv Z , Xu T , Li R , et al. Downregulation of m6A Methyltransferase in the hippocampus of Tyrobp^−/−^ mice and implications for learning and memory deficits. Front Neurosci. 2022;16:739201. 35386591 10.3389/fnins.2022.739201 PMC8978996

[26] Pupak A , Singh A , Sancho‐Balsells A , et al. Altered m6A RNA methylation contributes to hippocampal memory deficits in Huntington's disease mice. Cell Mol Life Sci. 2022;79(8):416. 35819730 10.1007/s00018-022-04444-6 PMC9276730

[27] Cai B , Song XQ , Cai JP , Zhang S . HOTAIR: a cancer‐related long non‐coding RNA. Neoplasma. 2014;61(4):379‐391. 25027739 10.4149/neo_2014_075

[28] Biswas S , Feng B , Chen S , et al. The long non‐coding RNA HOTAIR is a critical epigenetic mediator of angiogenesis in diabetic retinopathy. Invest Ophthalmol Vis Sci. 2021;62(3):20. 10.1167/iovs.62.3.20 PMC7980040 33724292

[29] Miao Z , Ding J , Chen B , Yang Y , Chen Y . HOTAIR overexpression correlated with worse survival in patients with solid tumors. Minerva Med. 2016;107(6):392‐400. 27333150

[30] Cui X , Pertile RAN , Du Z , et al. Developmental inhibition of long intergenic non‐coding RNA, HOTAIRM1, impairs dopamine neuron differentiation and maturation. Int J Mol Sci. 2021;22(14):7268. 34298885 10.3390/ijms22147268 PMC8306845

[31] Hu Q , Kim EJ , Feng J , Grant GR , Heller EA . Histone posttranslational modifications predict specific alternative exon subtypes in mammalian brain. PLoS Comput Biol. 2017;13(6):e1005602. 28609483 10.1371/journal.pcbi.1005602 PMC5487056

[32] Zhao M , Liang G , Wu X , et al. Abnormal epigenetic modifications in peripheral blood mononuclear cells from patients with alopecia areata. Br J Dermatol. 2012;166(2):226‐273. 10.1111/j.1365-2133.2011.10646.x 21936853

[33] Christopher MA , Myrick DA , Barwick BG , et al. LSD1 protects against hippocampal and cortical neurodegeneration. Nat Commun. 2017;8(1):805. 28993646 10.1038/s41467-017-00922-9 PMC5634471

[34] Chen M , Zhang X , Hao W . H3K4 dimethylation at FosB promoter in the striatum of chronic stressed rats promotes morphine‐induced conditioned place preference. PLoS One. 2019;14(8):e0221506. 31442272 10.1371/journal.pone.0221506 PMC6707596

[35] Kyzar EJ , Zhang H , Sakharkar AJ , Pandey SC . Adolescent alcohol exposure alters lysine demethylase 1 (LSD1) expression and histone methylation in the amygdala during adulthood. Addict Biol. 2017;22(5):1191‐1204. 27183824 10.1111/adb.12404 PMC5110402

