# Supplementary material for: METTL14‐mediated upregulation of lncRNA HOTAIR represses PP1α expression by promoting H3K4me1 demethylation in oxycodone‐treated mice
Source: CNS Neurosci Ther. 2024 Jul 24;30(7):e14830. doi: 10.1111/cns.14830 (PMC11267563; doi:10.1111/cns.14830)

Full unedited gel for Figure 3B: METTL14

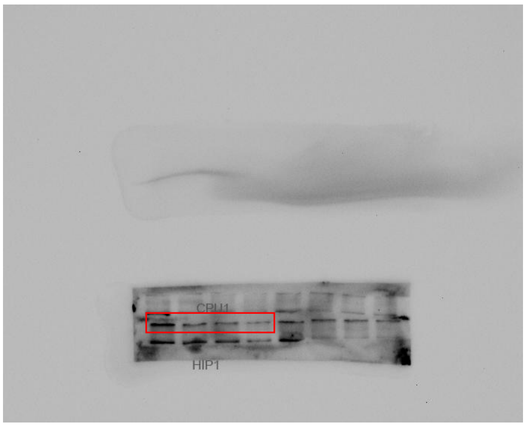

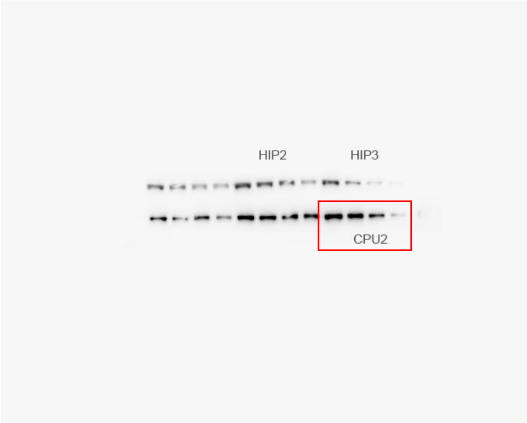

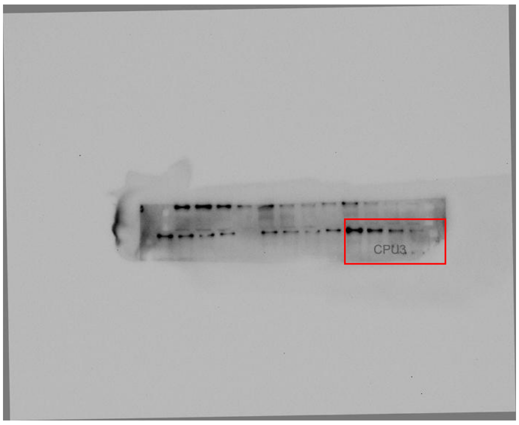

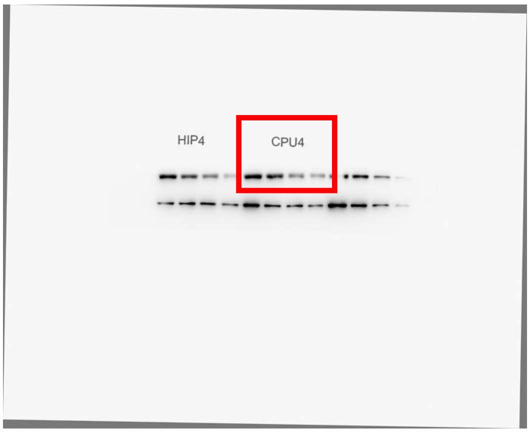

Full unedited gel for Figure 3B: PP1α

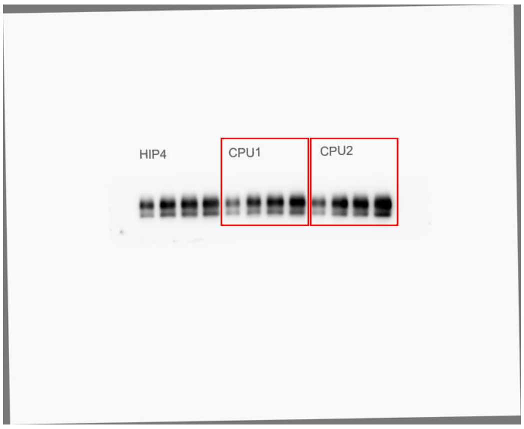

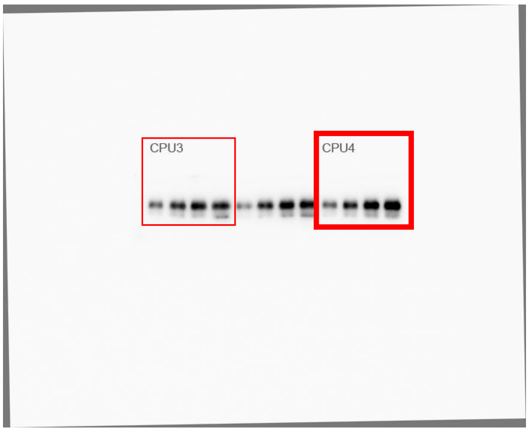

Full unedited gel for Figure 3F: METTL14

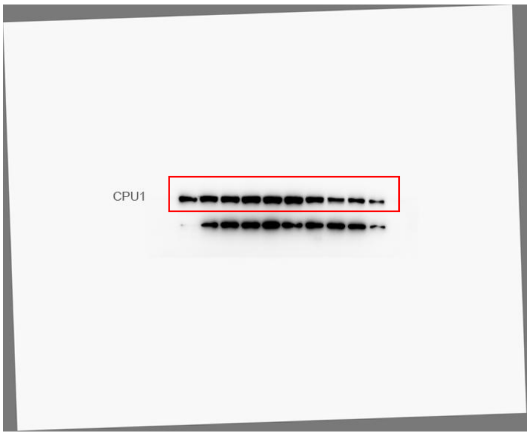

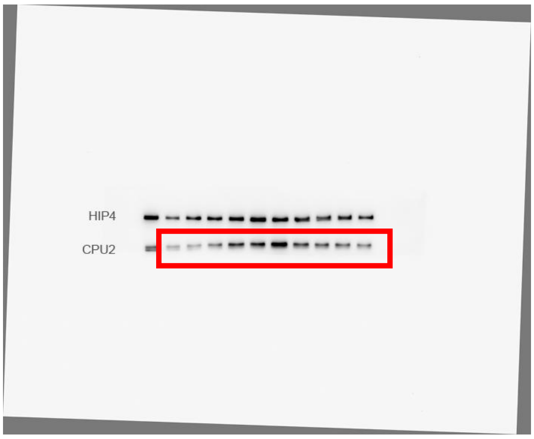

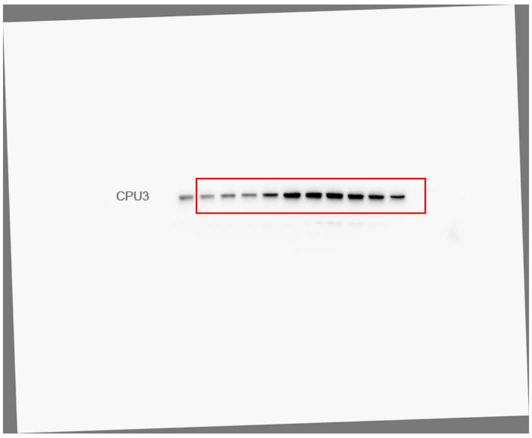

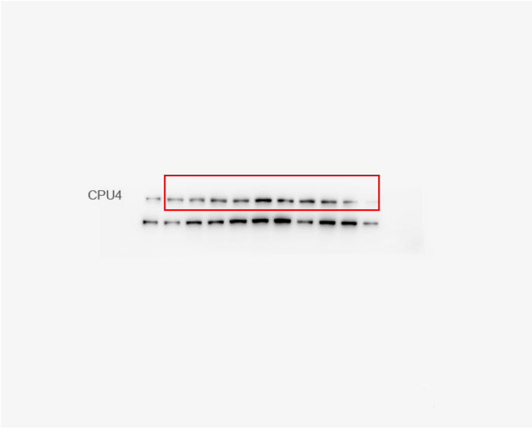

Full unedited gel for Figure 3F: PP1α

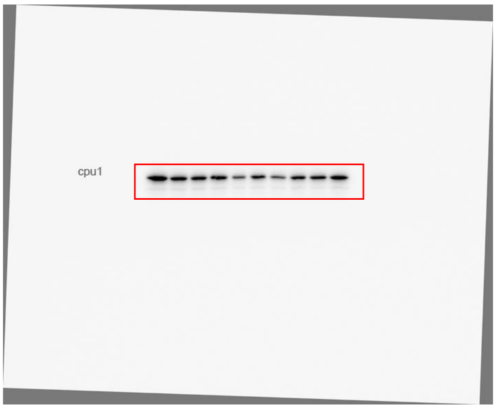

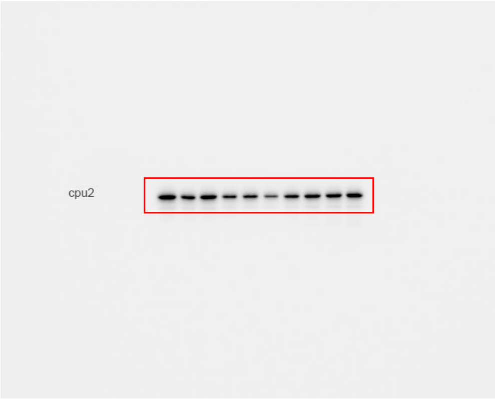

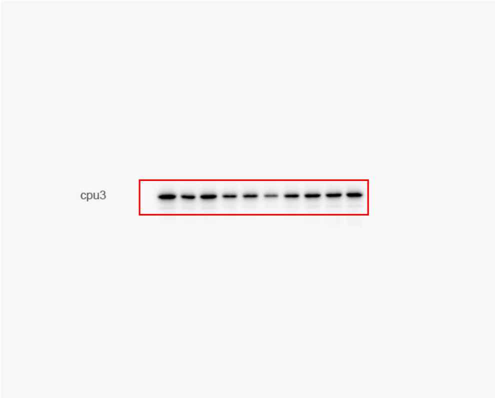

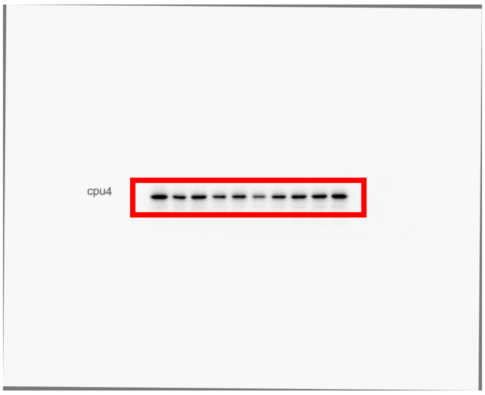

Full unedited gel for Figure 3B, 3F: β-actin

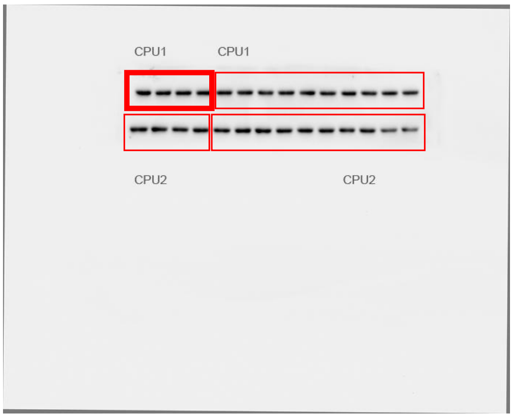

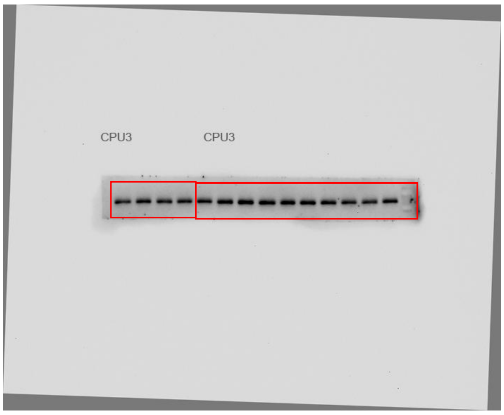

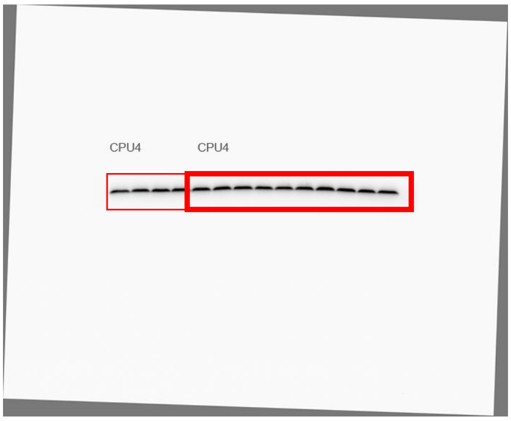

Full unedited gel for Figure 4A: METTL14

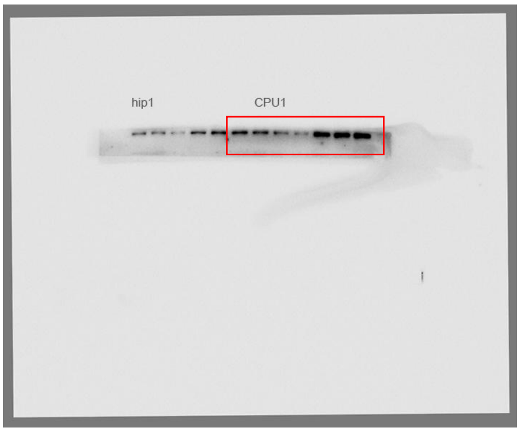

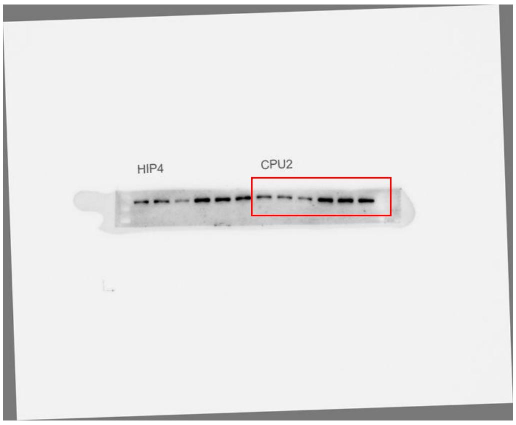

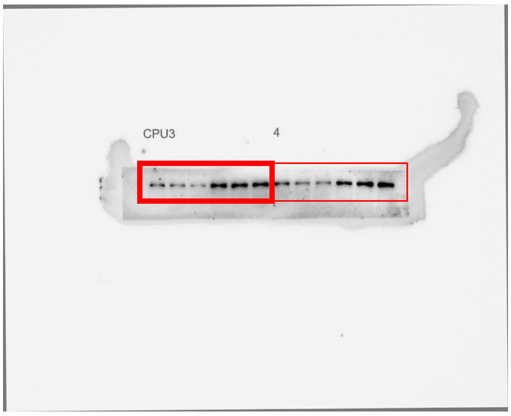

Full unedited gel for Figure 4A: β-actin

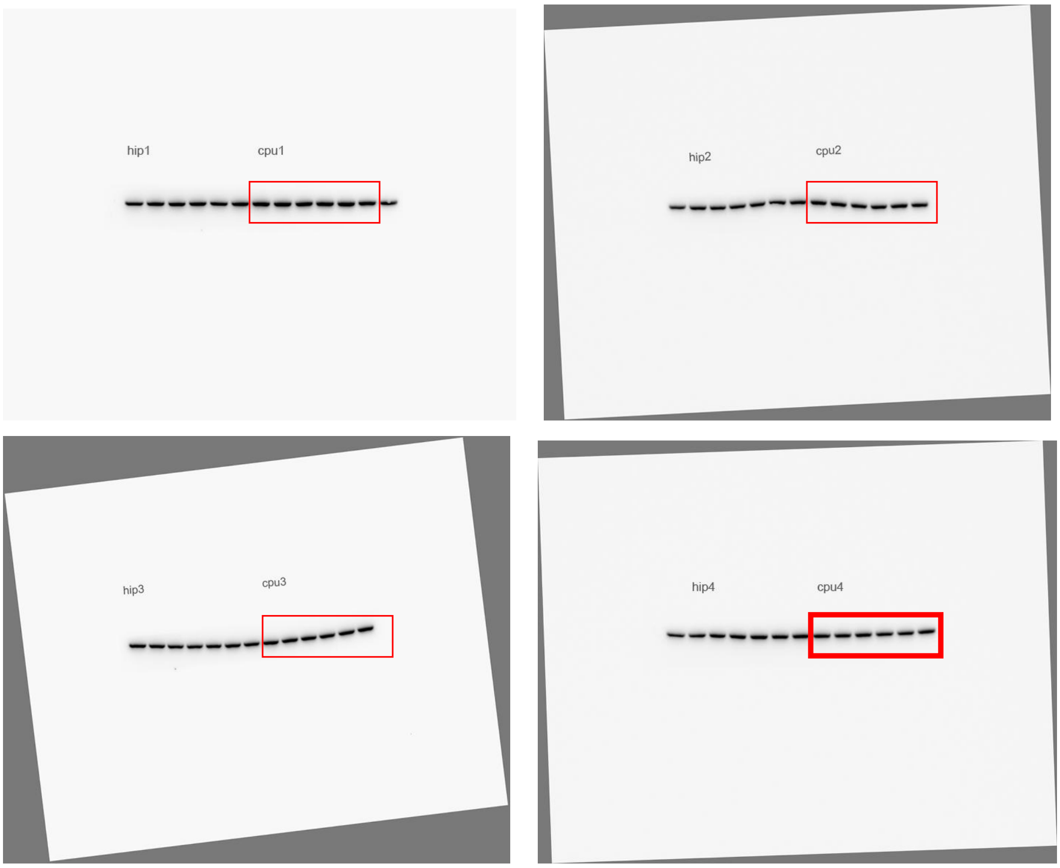

Full unedited gel for Figure 4D: METTL14

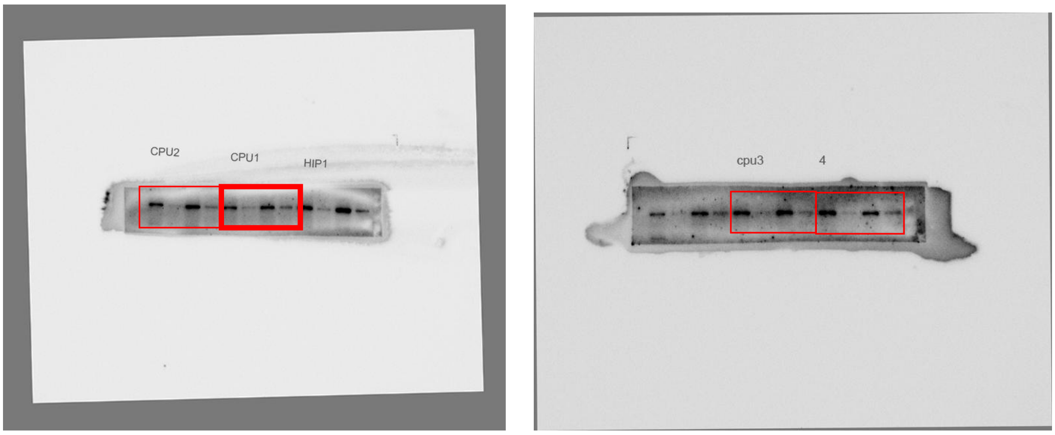

Full unedited gel for Figure 4D: PP1α

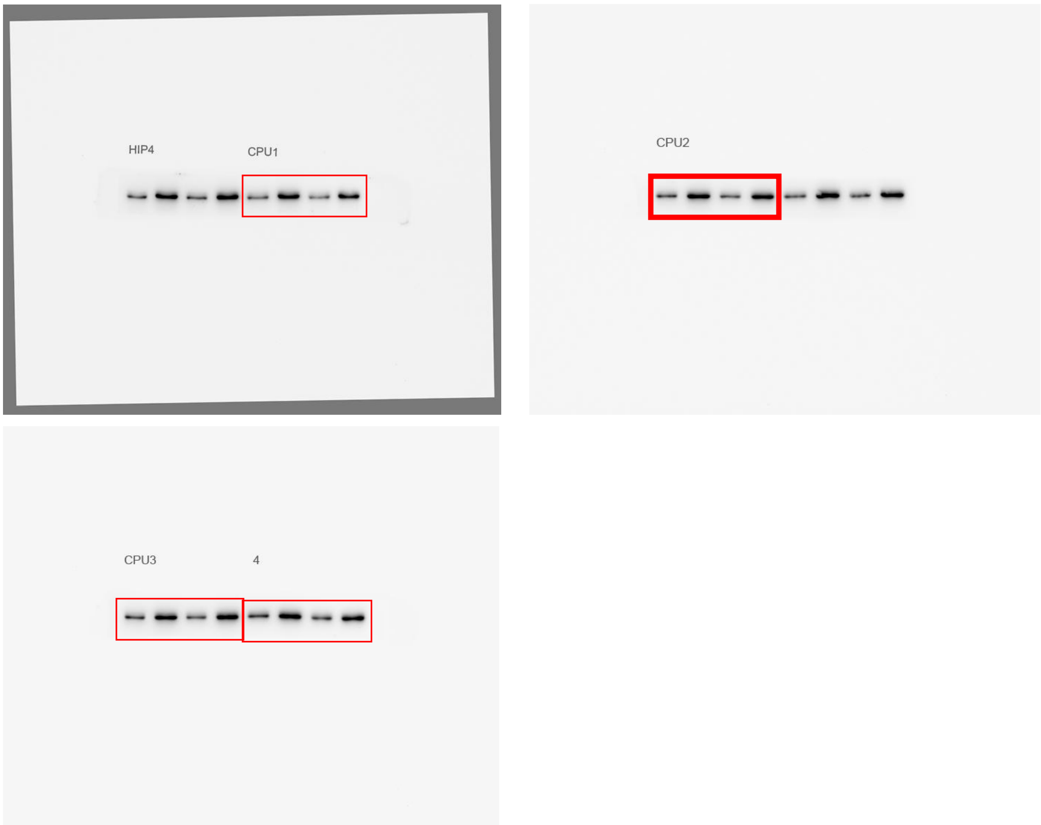

Full unedited gel for Figure 4D: β-actin

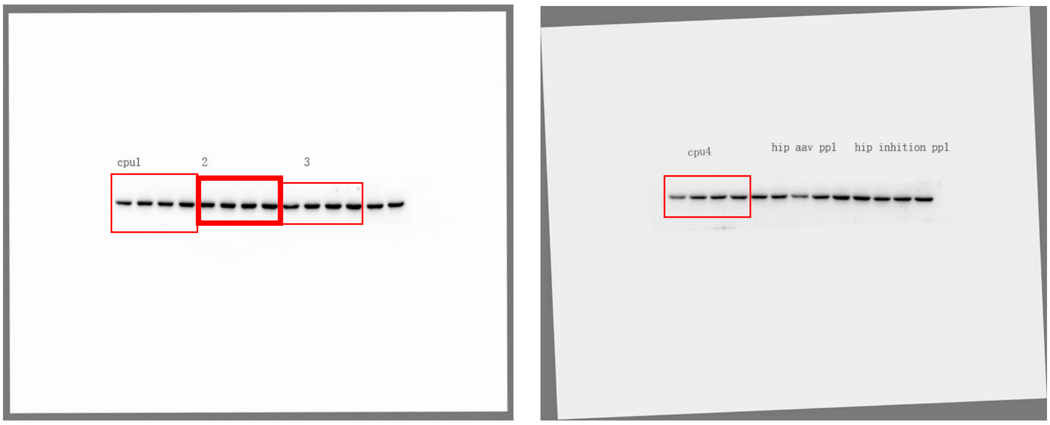

Full unedited gel for Figure 5D: PP1α

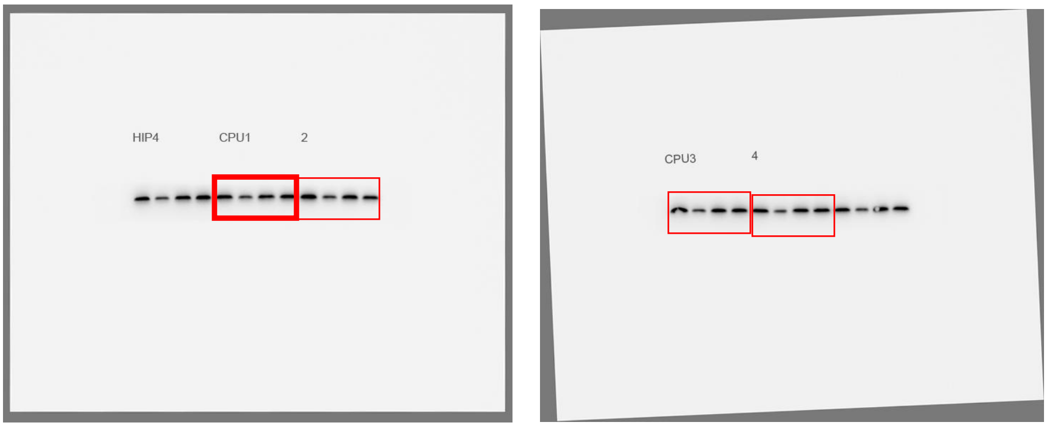

Full unedited gel for Figure 5D: β-actin

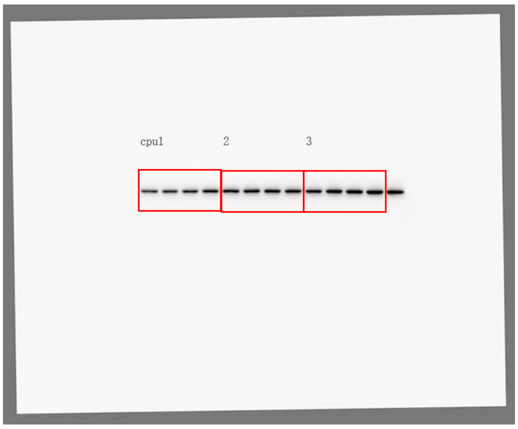

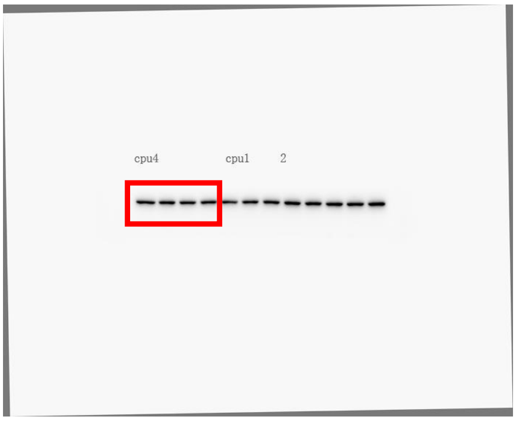

Full unedited gel for Figure 6C: LSD1

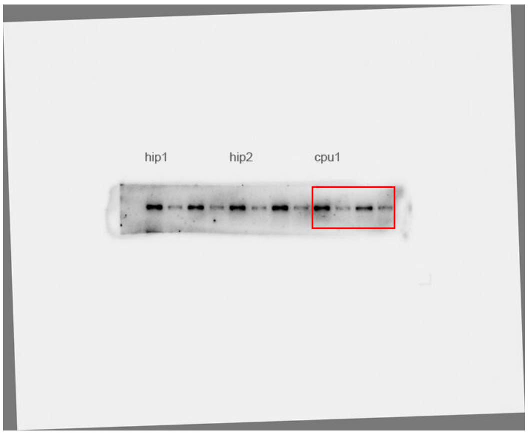

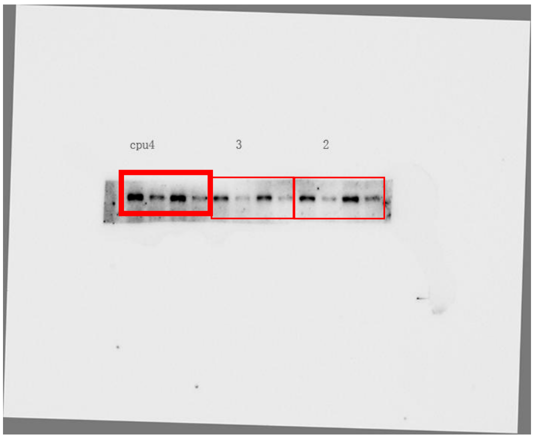

Full unedited gel for Figure 6C: PP1α

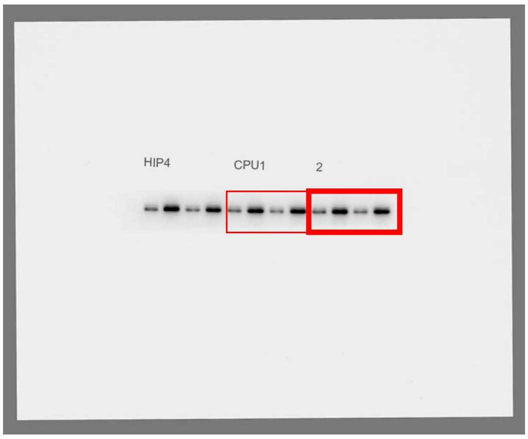

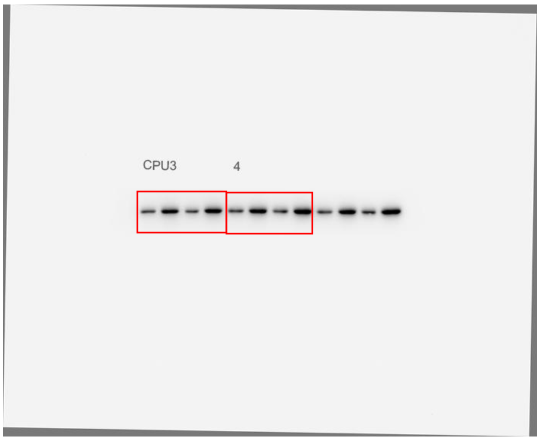

Full unedited gel for Figure 6C: β-actin

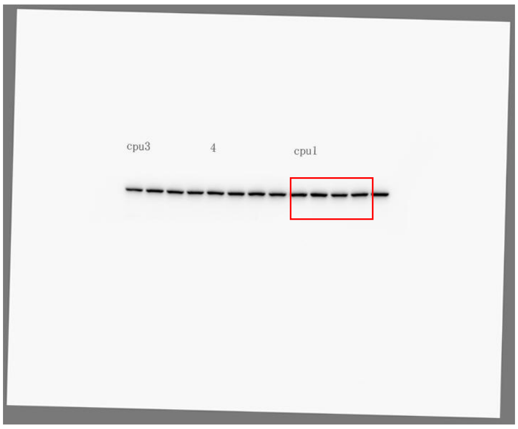

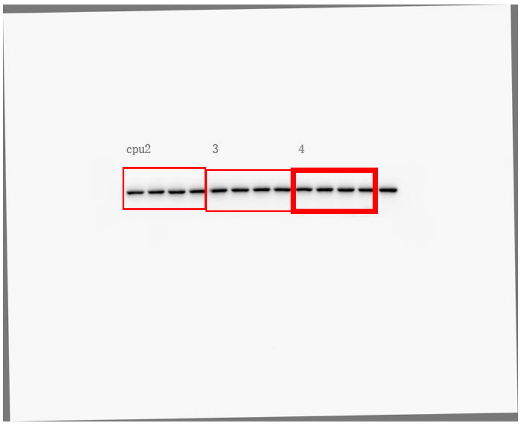

Full unedited gel for Figure 7A: H3K4me2

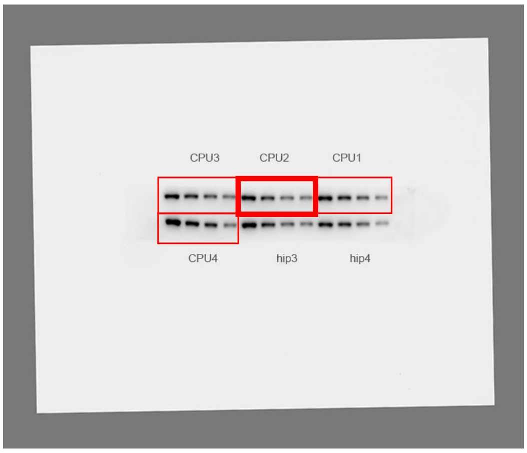

Full unedited gel for Figure 7A: CoRest

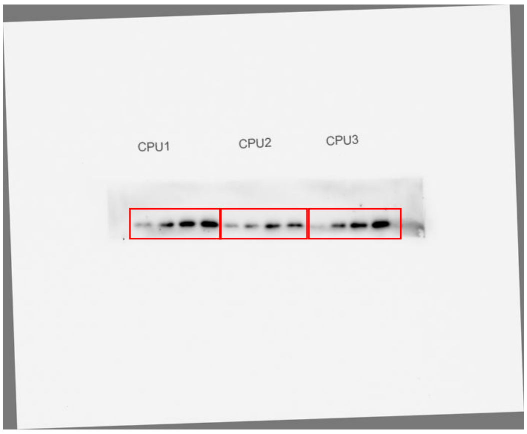

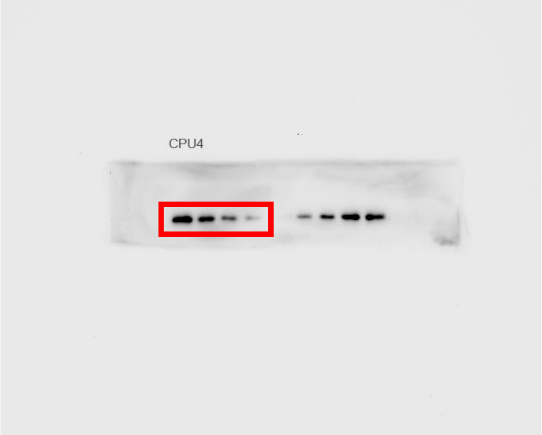

Full unedited gel for Figure 7A: H3K4me3

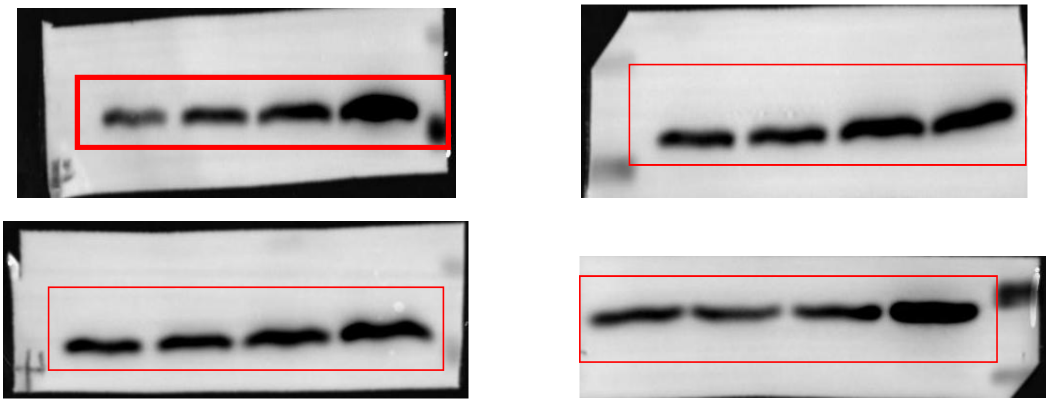

Full unedited gel for Figure 7A: H3K4me1

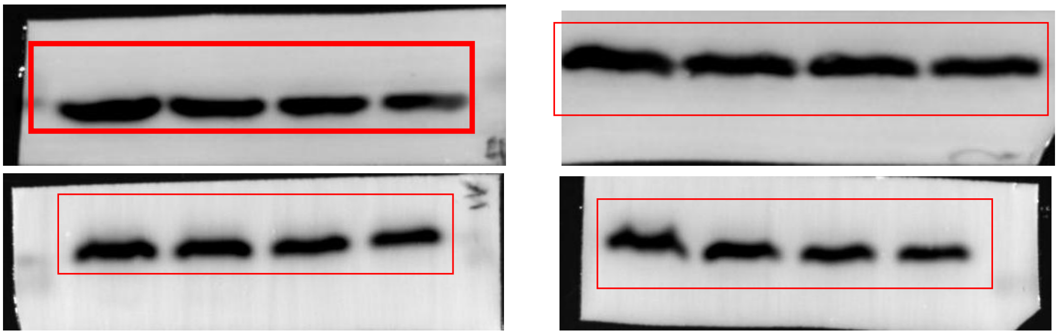

Full unedited gel for Figure 7A: KDM5A

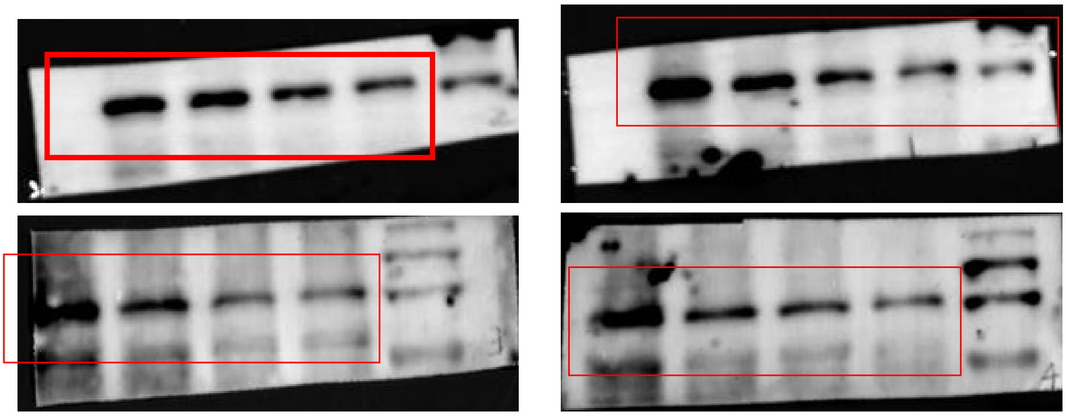

Full unedited gel for Figure 7G: H3K4me2

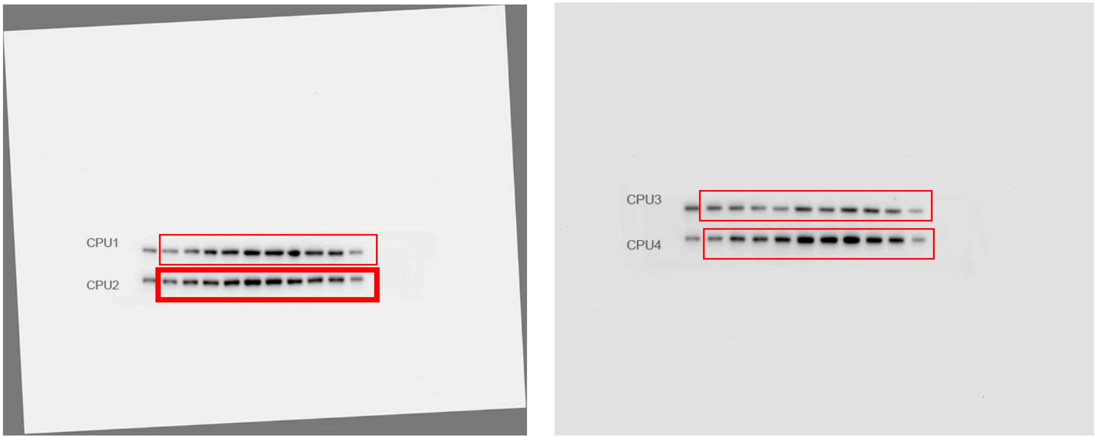

Full unedited gel for Figure 7G: CoRest

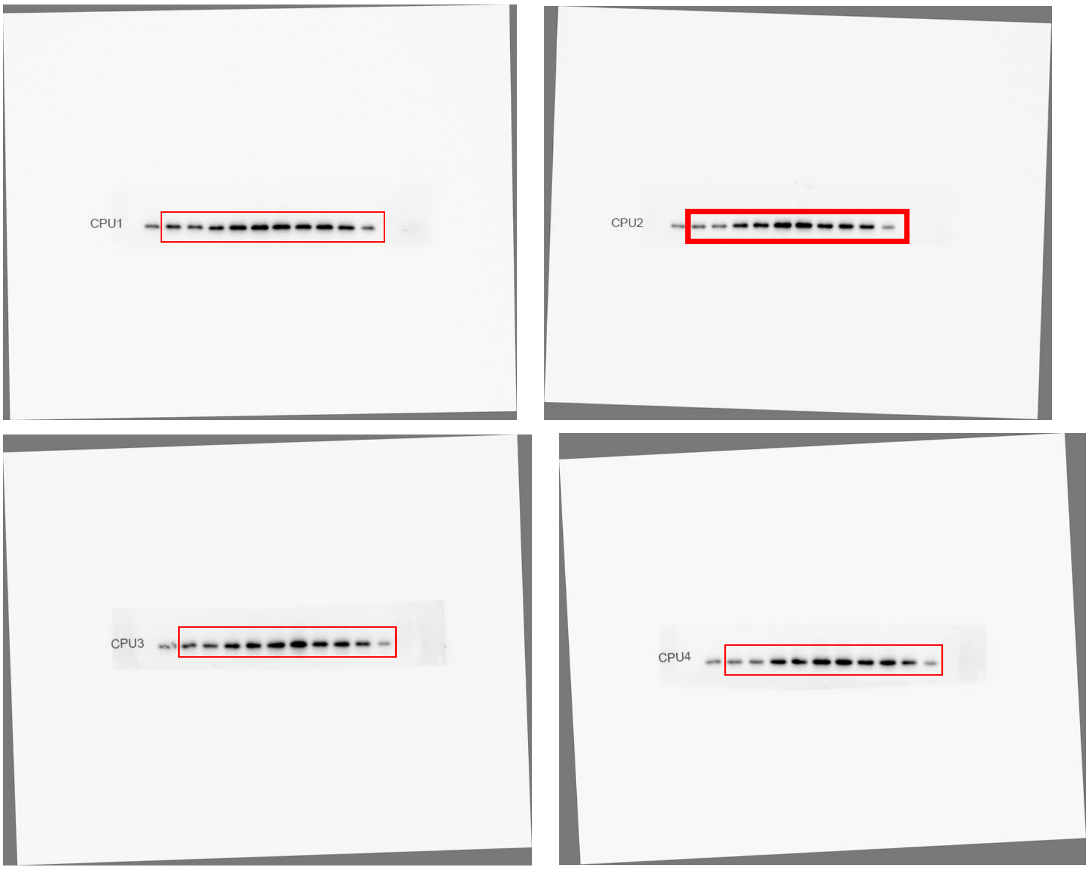

Full unedited gel for Figure 7G: H3K4me3

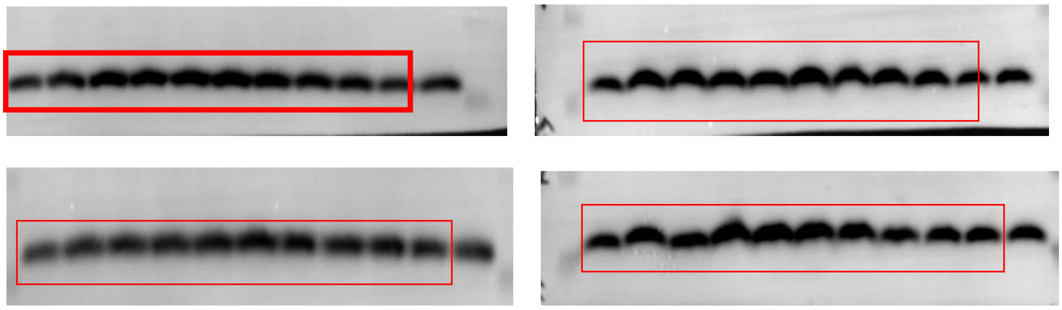

Full unedited gel for Figure 7G: H3K4me1

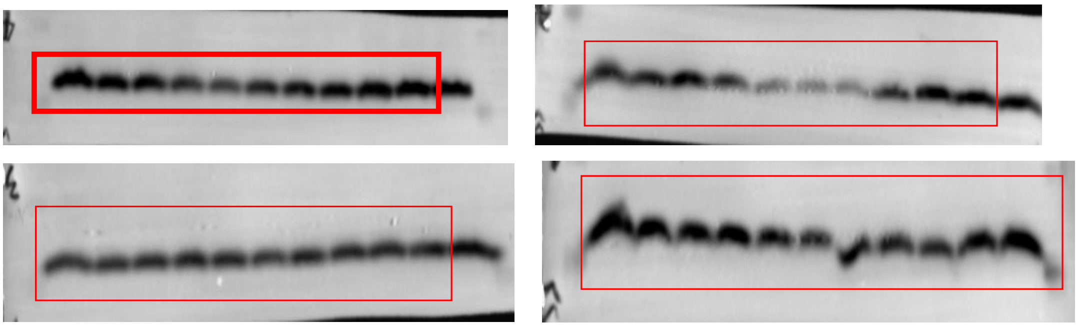

Full unedited gel for Figure 7A, 7G: H3

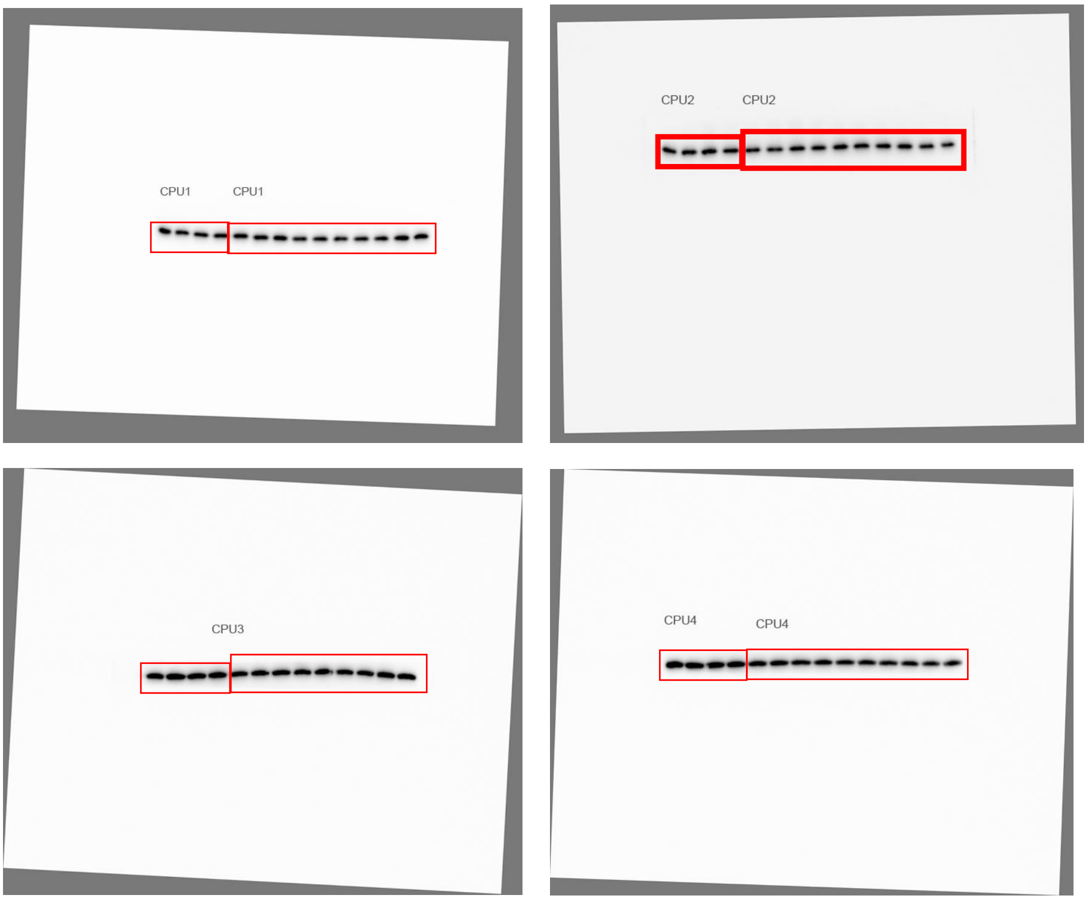

Full unedited gel for Figure 7L: H3K4me1

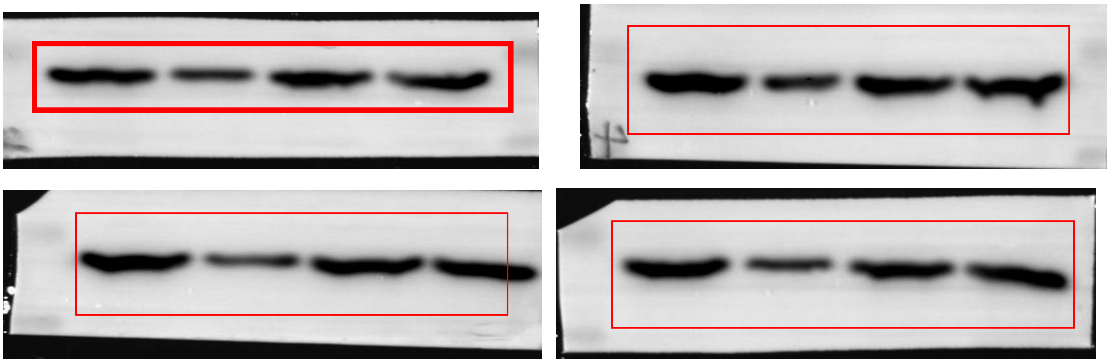

Full unedited gel for Figure 7L: H3K4me3

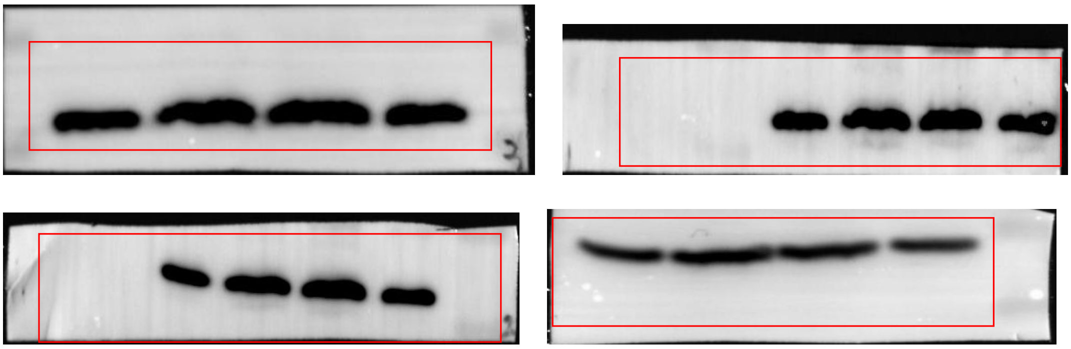

Full unedited gel for Figure 7L: H3K4me2

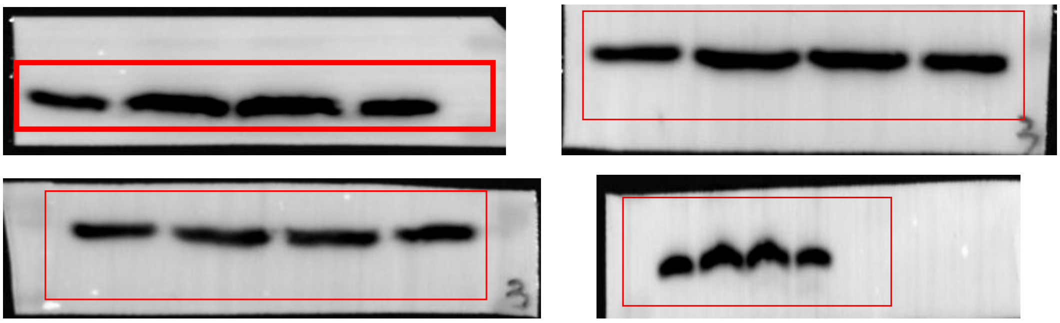

Full unedited gel for Figure 7L: H3

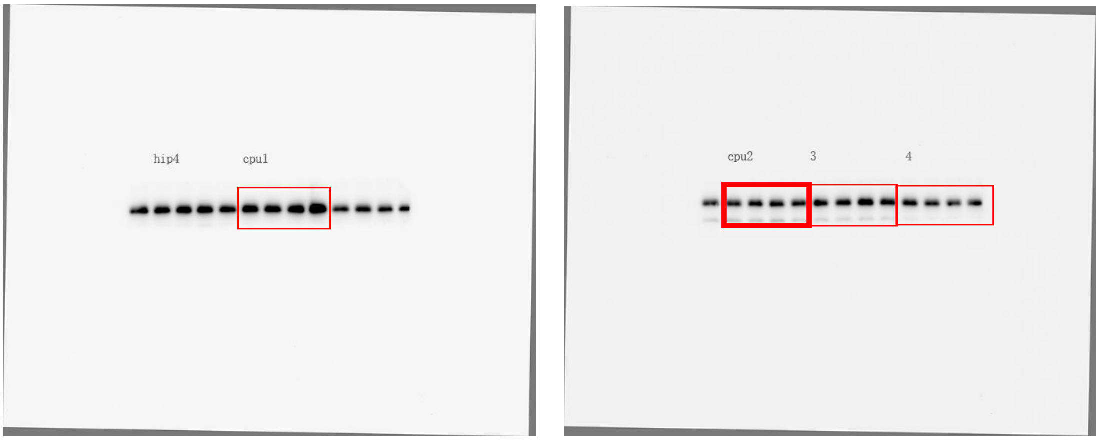

Full unedited gel for Figure 7N

LSD1 (input):

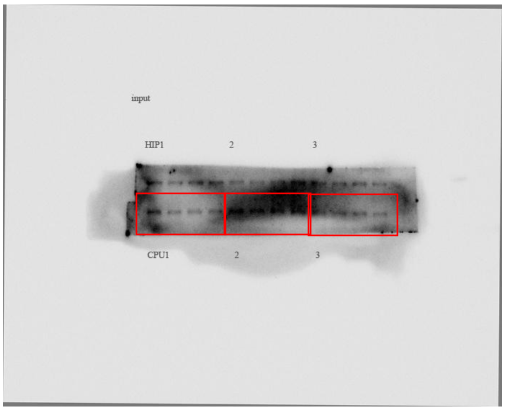

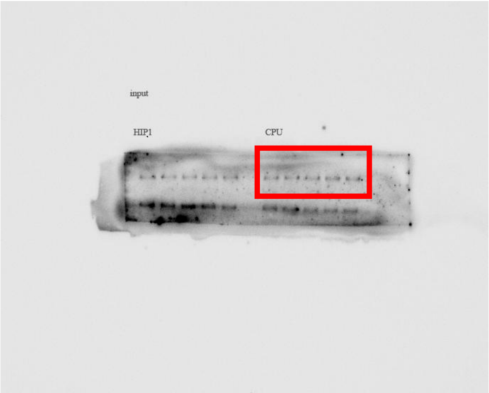

CoRest (input):

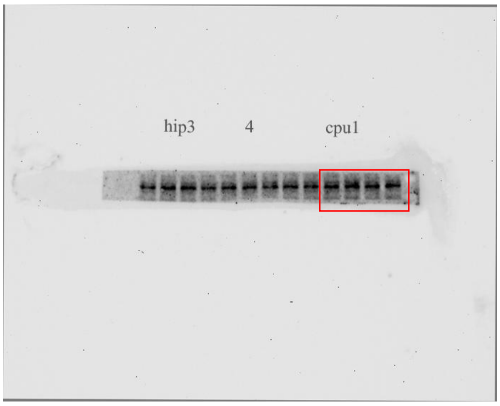

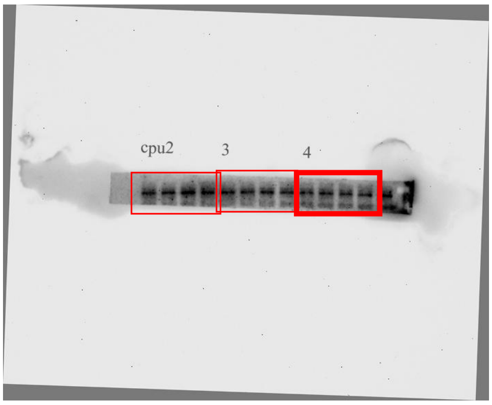

LSD1 (IB):

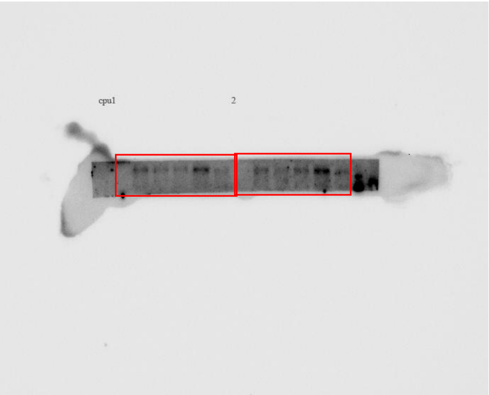

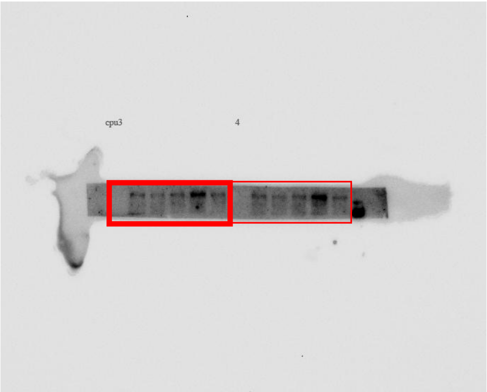

CoRest (IB):

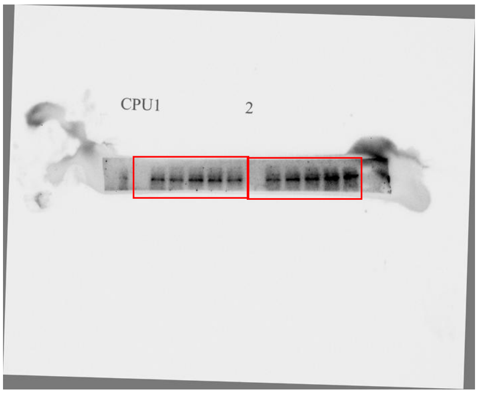

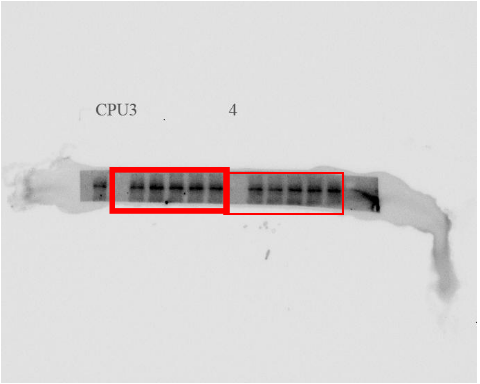

Supplement: Supplementary file 1 — DataS1 [file CNS-30-e14830-s002.pdf]
