# Supplementary figures and images for: METTL14‐mediated upregulation of lncRNA HOTAIR represses PP1α expression by promoting H3K4me1 demethylation in oxycodone‐treated mice
Source: CNS Neurosci Ther. 2024 Jul 24;30(7):e14830. doi: 10.1111/cns.14830 (PMC11267563; doi:10.1111/cns.14830)

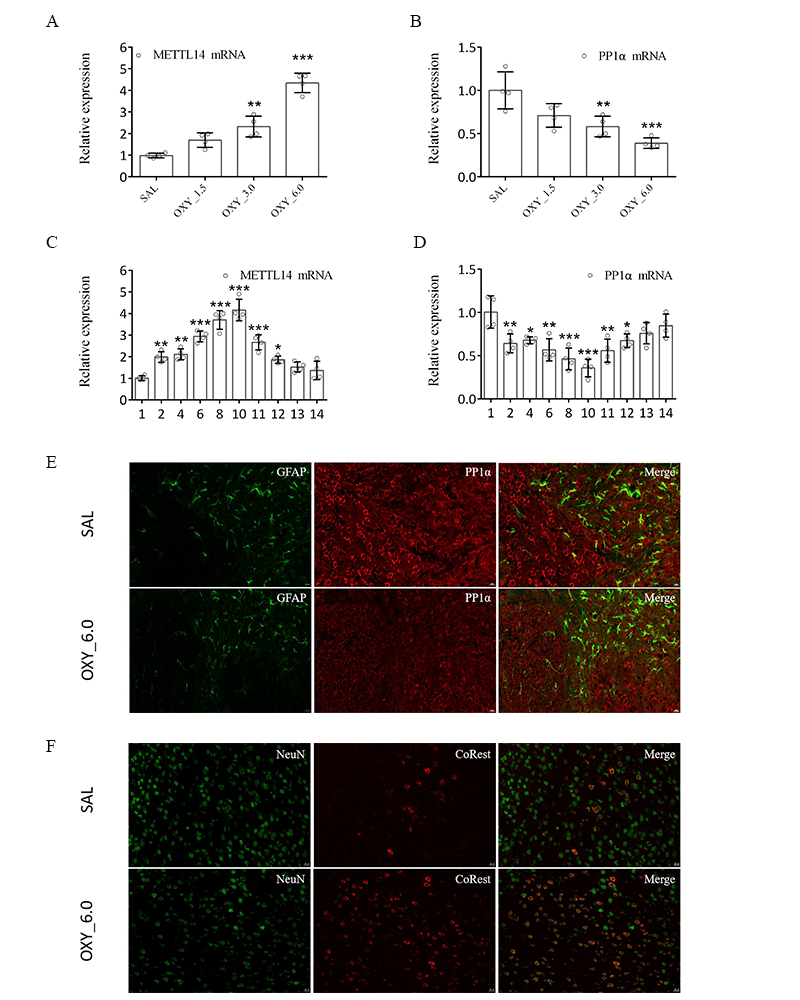

Supplement: Supplementary file 2 — FigureS1 [file CNS-30-e14830-s003.tif]
