# Supplementary material for: METTL14‐mediated upregulation of lncRNA HOTAIR represses PP1α expression by promoting H3K4me1 demethylation in oxycodone‐treated mice
Source: CNS Neurosci Ther. 2024 Jul 24;30(7):e14830. doi: 10.1111/cns.14830 (PMC11267563; doi:10.1111/cns.14830)
Supplement: Supplementary file 3 — TableS1 [file CNS-30-e14830-s001.docx]

Table 1. The comparison of differential genes expression between the OXY and SAL group

| Type | Gene ID | Gene Symbol | log2(OXY/SAL) | Qvalue(OXY/SAL) |
| --- | --- | --- | --- | --- |
| mRNA | 100039192 | 'Tmem254c' | -1.71516 | 7.53E-30 |
|  | 100039293 | 'Duxbl3' | -2.64964 | 9.59E-08 |
|  | 100039863 | 'Gm13306' | 1.159228 | 1.85E-04 |
|  | 100042100 | 'Gm3667' | -1.21929 | 2.14E-05 |
|  | 100302688 | 'Gm17455' | -1.59585 | 1.41E-04 |
|  | 100503710 | 'Gm5741' | -2.08979 | 9.74E-07 |
|  | 101055676 | 'LOC101055676' | 1.289952 | 9.29E-08 |
|  | 101056159 | 'LOC101056159' | -2.19611 | 2.01E-04 |
|  | 101488212 | 'Evi2' | -1.95563 | 8.47E-05 |
|  | 104383 | 'Rcor2' | 1.220221 | 1.13E-05 |
|  | 108015 | 'Chrnb4' | -1.51084 | 4.69E-38 |
|  | 110312 | 'Pmch' | -5.06561 | 3.16E-210 |
|  | 110834 | 'Chrna3' | -1.43917 | 1.06E-52 |
|  | 114654 | 'Ly6g6d' | -1.3048 | 9.75E-04 |
|  | 11540 | 'Adora2a' | -1.53275 | 1.04E-23 |
|  | 118449 | 'Synpo2' | -1.00154 | 2.67E-254 |
|  | 12311 | 'Calcr' | 1.860404 | 2.08E-06 |
|  | 12504 | 'Cd4' | -1.62333 | 9.46E-05 |
|  | 12587 | 'Mia' | -1.07944 | 2.26E-06 |
|  | 12647 | 'Chat' | -1.51339 | 1.46E-20 |
|  | 13489 | 'Drd2' | -1.03679 | 7.99E-25 |
|  | 13507 | 'Dsc3' | -2.13612 | 4.71E-15 |
|  | 13835 | 'Epha1' | -1.03598 | 7.18E-04 |
|  | 140491 | 'Ppp1r3a' | -1.15594 | 6.14E-06 |
|  | 140741 | 'Gpr6' | -1.17789 | 8.72E-05 |
|  | 14160 | 'Lgr5' | -1.19109 | 8.05E-22 |
|  | 14599 | 'Gh' | -7.61587 | 2.15E-85 |
|  | 14654 | 'Glra1' | -1.00339 | 1.63E-26 |
|  | 14917 | 'Gucy2c' | -1.45236 | 2.27E-05 |
|  | 15139 | 'Hc' | -2.38497 | 2.19E-05 |
|  | 15171 | 'Hcrt' | -8.03272 | 4.81E-55 |
|  | 15375 | 'Foxa1' | -2.1528 | 9.98E-05 |
|  | 15552 | 'Htr1d' | -1.28243 | 7.51E-06 |
|  | 16370 | 'Irs4' | 1.943963 | 2.46E-35 |
|  | 16371 | 'Irx1' | -1.28144 | 7.06E-19 |
|  | 16372 | 'Irx2' | -1.03151 | 1.80E-14 |
|  | 16373 | 'Irx3' | -1.15765 | 1.64E-07 |
|  | 16869 | 'Lhx1' | -1.11614 | 1.46E-06 |
|  | 16917 | 'Lmx1b' | -1.53541 | 8.33E-04 |
|  | 17116 | 'Mab21l1' | -1.26103 | 9.15E-12 |
|  | 171211 | 'Edaradd' | -1.0258 | 2.95E-12 |
|  | 17349 | 'Mlf1' | -1.25296 | 6.06E-05 |
|  | 18053 | 'Ngfr' | -1.35838 | 2.22E-23 |
|  | 18429 | 'Oxt' | 4.195932 | 1.57E-35 |
|  | 18741 | 'Pitx2' | -3.91361 | 5.41E-13 |
|  | 18753 | 'Prkcd' | -1.04278 | 0 |
|  | 18976 | 'Pomc' | -1.4423 | 2.35E-06 |
|  | 18996 | 'Pou4f1' | -1.55901 | 3.01E-36 |
|  | 19109 | 'Prl' | -2.47764 | 6.46E-09 |
|  | 19132 | 'Prph' | -1.19047 | 2.49E-05 |
|  | 19144 | 'Klk6' | -1.88895 | 3.51E-43 |
|  | 19734 | 'Rgs16' | -1.05612 | 4.60E-170 |
|  | 20429 | 'Shox2' | -1.00612 | 2.45E-74 |
|  | 20473 | 'Six3' | -1.24267 | 3.43E-28 |
|  | 20508 | 'Slc18a3' | -1.72414 | 1.83E-19 |
|  | 208613 | 'Tmem212' | -1.03123 | 1.35E-07 |
|  | 208898 | 'Unc13c' | -1.22338 | 0 |
|  | 209776 | 'Gpr139' | -1.09374 | 5.46E-16 |
|  | 210529 | Mettl14' | 2.061523 | 3.09E-04 |
|  | 211623 | 'Plac9a' | -1.23705 | 9.02E-07 |
|  | 21334 | 'Tac2' | -1.16713 | 1.82E-38 |
|  | 21416 | 'Tcf7l2' | -1.09227 | 0 |
|  | 217143 | 'Gpr179' | -1.21147 | 6.15E-11 |
|  | 21818 | 'Tgm3' | 1.320521 | 2.68E-07 |
|  | 21955 | 'Tnnt1' | -1.21644 | 4.22E-48 |
|  | 22295 | 'Cdh23' | -1.31525 | 7.14E-32 |
|  | 22355 | 'Vipr2' | -1.06127 | 3.12E-33 |
|  | 22402 | 'Ccn4' | 1.102211 | 2.04E-10 |
|  | 22445 | 'Xlr3a' | -2.38101 | 7.76E-16 |
|  | 225638 | 'Alpk2' | -1.01682 | 6.94E-04 |
|  | 22626 | 'Slc23a3' | 1.121466 | 1.77E-04 |
|  | 226438 | 'Igfn1' | 1.043779 | 5.56E-72 |
|  | 228911 | 'Tshz2' | -1.00984 | 0 |
|  | 237987 | 'Otop2' | 1.664827 | 1.81E-04 |
|  | 240239 | 'Gpr151' | -1.42455 | 2.94E-48 |
|  | 240892 | 'Dusp27' | -1.05742 | 3.47E-06 |
|  | 24117 | 'Wif1' | -1.27926 | 3.42E-08 |
|  | 243369 | 'Sspo' | -1.10225 | 1.29E-50 |
|  | 269328 | 'Muc15' | -1.79023 | 8.95E-04 |
|  | 278507 | 'Wfikkn2' | -1.06169 | 1.81E-16 |
|  | 319508 | 'Syt15' | -1.04989 | 1.80E-08 |
|  | 329375 | 'Cfap77' | -1.60178 | 6.29E-04 |
|  | 329482 | 'Dcdc5' | -1.09835 | 2.14E-14 |
|  | 333883 | 'Cd59b' | -1.22725 | 3.70E-04 |
|  | 380683 | 'Sec14l3' | -1.41583 | 9.30E-06 |
|  | 381409 | 'Cdh26' | -1.29698 | 1.01E-05 |
|  | 384198 | 'Fam47e' | -1.28055 | 7.30E-04 |
|  | 386454 | 'Rnf39' | 1.028534 | 2.06E-10 |
|  | 433182 | 'Eno1b' | -1.82609 | 3.69E-05 |
|  | 434794 | 'Xlr4a' | -2.69754 | 1.60E-07 |
|  | 50905 | 'Il17rb' | -1.047 | 3.66E-05 |
|  | 54352 | 'Irx5' | -1.46234 | 3.15E-10 |
|  | 54612 | 'Sfrp5' | -1.14685 | 1.28E-11 |
|  | 56523 | 'Pmfbp1' | -1.9366 | 9.45E-07 |
|  | 574437 | 'Xlr3b' | -1.62243 | 9.69E-18 |
|  | 58222 | 'Rab37' | -1.05631 | 6.87E-65 |
|  | 60344 | 'Fign' | -1.03685 | 2.66E-92 |
|  | 60510 | 'Syt9' | -1.10527 | 7.27E-209 |
|  | 626359 | 'Wdr93' | -1.1864 | 2.60E-04 |
|  | 63993 | 'Slc5a7' | -1.41377 | 8.28E-45 |
|  | 68553 | 'Col6a4' | 2.108706 | 1.35E-49 |
|  | 68810 | 'Nexn' | -1.23853 | 1.14E-30 |
|  | 74190 | 'Exoc3l4' | -1.07942 | 7.67E-11 |
|  | 75563 | 'Dnali1' | -1.00811 | 5.17E-07 |
|  | 76113 | 'Lpo' | -1.19968 | 7.40E-05 |
|  | 99982 | 'Kdm1a' | 3.140729 | 5.92E-04 |
| lncRNA | 100503872 | 'Hotair' | 1.001255 | 5.48E-44 |
|  | 100504399 | 'C130021I20Rik' | -1.87269 | 1.40E-06 |
|  | 102634887 | 'Gm13112' | -1.28243 | 2.69E-09 |
|  | 104798 | 'Sfta3-ps' | -1.84846 | 9.06E-14 |
|  | 319970 | 'B230323A14Rik' | -1.36138 | 1.59E-04 |
|  | 320155 | 'C730002L08Rik' | 1.79157 | 1.77E-06 |
|  | 320249 | 'D130009I18Rik' | -1.4423 | 3.11E-04 |
|  | 320614 | 'A330033J07Rik' | -1.00305 | 6.60E-06 |
|  | 328287 | 'Gm20554' | -1.35074 | 3.00E-21 |
|  | 622208 | 'Gm6297' | 6.718401 | 1.59E-12 |
|  | 71296 | 'Crnde' | -1.4568 | 3.17E-07 |
|  | URS0000453061 | 'URS0000453061' | -10.2143 | 5.56E-10 |
|  | URS000076DC1A | 'URS000076DC1A' | 1.667293 | 3.68E-28 |
|  | URS0000775AAA | 'URS0000775AAA' | 2.197735 | 5.20E-04 |
|  | URS00007890D9 | 'URS00007890D9' | 1.386047 | 5.78E-11 |
|  | URS00008C41CF | 'URS00008C41CF' | 5.068501 | 1.73E-04 |
|  | URS00009AD0FC | 'URS00009AD0FC' | 6.001719 | 4.79E-08 |
|  | URS00009AD3E3 | 'URS00009AD3E3' | -1.36592 | 3.34E-04 |
|  | URS00009AD6FF | 'URS00009AD6FF' | -1.43368 | 9.50E-09 |
|  | URS00009AE0D9 | 'URS00009AE0D9' | -1.35633 | 4.86E-04 |
|  | URS00009AEC10 | 'URS00009AEC10' | 2.229224 | 1.11E-09 |
|  | URS00009AED7B | 'URS00009AED7B' | 2.922557 | 1.22E-04 |
|  | URS00009AF071 | 'URS00009AF071' | -1.96457 | 2.27E-05 |
|  | URS00009AF72F | 'URS00009AF72F' | -1.44184 | 3.69E-05 |
|  | URS00009AFBFA | 'URS00009AFBFA' | 2.542412 | 9.46E-05 |
|  | URS00009B003F | 'URS00009B003F' | 3.063423 | 2.03E-06 |
|  | URS00009B0CA7 | 'URS00009B0CA7' | 1.454193 | 2.49E-05 |
|  | URS00009B0DC0 | 'URS00009B0DC0' | 2.21442 | 2.22E-06 |
|  | URS00009B143E | 'URS00009B143E' | 1.048156 | 2.11E-04 |
|  | URS00009B147E | 'URS00009B147E' | -1.81189 | 1.52E-08 |
|  | URS00009B2941 | 'URS00009B2941' | -2.93983 | 4.73E-05 |
|  | URS00009B2F10 | 'URS00009B2F10' | -4.83087 | 6.55E-04 |
|  | URS00009B3573 | 'URS00009B3573' | -2.06976 | 4.33E-06 |
|  | URS00009B4A8F | 'URS00009B4A8F' | -4.79023 | 8.18E-04 |
|  | URS00009B4DB4 | 'URS00009B4DB4' | 2.537041 | 2.33E-07 |
|  | URS00009B52DF | 'URS00009B52DF' | 1.758854 | 2.28E-07 |
|  | URS00009B5324 | 'URS00009B5324' | -4.88301 | 4.86E-04 |
|  | URS00009B546F | 'URS00009B546F' | 1.466236 | 7.37E-11 |
|  | URS00009B5A73 | 'URS00009B5A73' | 1.28315 | 8.30E-04 |
|  | URS00009B5A81 | 'URS00009B5A81' | -2.89274 | 9.79E-04 |
|  | URS00009B5BA2 | 'URS00009B5BA2' | 4.906603 | 4.65E-04 |
|  | URS00009B6728 | 'URS00009B6728' | -1.12019 | 1.07E-04 |
|  | URS00009B708A | 'URS00009B708A' | 1.803169 | 1.77E-10 |
|  | URS00009B73A2 | 'URS00009B73A2' | -1.09722 | 2.45E-06 |
|  | URS00009B7AC7 | 'URS00009B7AC7' | -5.82081 | 2.88E-07 |
|  | URS00009B7F46 | 'URS00009B7F46' | -5.45055 | 5.76E-12 |
|  | URS00009B807E | 'URS00009B807E' | -2.22115 | 5.38E-04 |
|  | URS00009B8154 | 'URS00009B8154' | -3.27479 | 3.31E-04 |
|  | URS00009B8218 | 'URS00009B8218' | -1.65523 | 8.47E-07 |
|  | URS00009B8B36 | 'URS00009B8B36' | 1.26237 | 5.77E-12 |
|  | URS00009B8C63 | 'URS00009B8C63' | -1.54542 | 1.06E-04 |
|  | URS00009B8FAA | 'URS00009B8FAA' | -5.04205 | 1.85E-04 |
|  | URS00009B9AEC | 'URS00009B9AEC' | -4.82182 | 6.89E-04 |
|  | URS00009B9ED1 | 'URS00009B9ED1' | 4.867128 | 5.83E-04 |
|  | URS00009BA3FA | 'URS00009BA3FA' | -1.06048 | 1.21E-06 |
|  | URS00009BA942 | 'URS00009BA942' | -1.15198 | 4.16E-05 |
|  | URS00009BAC76 | 'URS00009BAC76' | -2.25827 | 2.28E-05 |
|  | URS00009BAF62 | 'URS00009BAF62' | -2.10403 | 1.90E-16 |
|  | URS00009BB40C | 'URS00009BB40C' | -5.73187 | 7.17E-07 |
|  | URS00009BBFDA | 'URS00009BBFDA' | 1.171574 | 3.22E-11 |
|  | URS00009BCCCA | 'URS00009BCCCA' | -1.23963 | 3.33E-04 |
|  | URS00009BE215 | 'URS00009BE215' | 6.995325 | 7.91E-15 |
|  | URS00009BEDB6 | 'URS00009BEDB6' | -3.12977 | 2.32E-06 |
|  | URS00009BEFAA | 'URS00009BEFAA' | 1.077439 | 7.51E-04 |
|  | URS00009BF022 | 'URS00009BF022' | 1.317406 | 2.55E-06 |
|  | URS00009BFD9F | 'URS00009BFD9F' | 3.229275 | 3.15E-04 |
|  | URS00009C0A99 | 'URS00009C0A99' | 1.028375 | 4.63E-98 |
|  | URS00009C0CC4 | 'URS00009C0CC4' | -4.95923 | 3.11E-04 |
|  | URS00009C0F26 | 'URS00009C0F26' | -5.46055 | 8.55E-06 |
|  | URS00009C129A | 'URS00009C129A' | 4.73889 | 7.12E-10 |
|  | URS00009C16F3 | 'URS00009C16F3' | -2.15966 | 5.15E-04 |
|  | URS00009C1725 | 'URS00009C1725' | 1.511228 | 8.13E-05 |
|  | URS00009C20CA | 'URS00009C20CA' | -5.58931 | 2.76E-06 |
|  | URS00009C2463 | 'URS00009C2463' | -4.33297 | 8.69E-04 |
|  | URS00009C37B0 | 'URS00009C37B0' | -1.46739 | 1.85E-04 |
|  | URS00009C45F4 | 'URS00009C45F4' | -1.0612 | 1.68E-07 |
|  | URS00009C4818 | 'URS00009C4818' | -1.39357 | 8.17E-05 |
|  | URS00009C5106 | 'URS00009C5106' | -1.64154 | 4.37E-04 |
|  | URS00009C5278 | 'URS00009C5278' | -5.02639 | 2.04E-04 |
|  | URS00009C54E4 | 'URS00009C54E4' | -5.70807 | 9.03E-07 |
|  | URS00009C5636 | 'URS00009C5636' | 4.782664 | 9.28E-04 |
|  | URS00009C56A1 | 'URS00009C56A1' | 5.08494 | 1.55E-04 |
|  | URS00009C5724 | 'URS00009C5724' | 5.466691 | 9.34E-06 |
|  | URS00009C5A92 | 'URS00009C5A92' | 1.691729 | 9.79E-06 |
|  | URS0000A8E7F0 | 'URS0000A8E7F0' | 12.95643 | 5.18E-16 |
|  | URS0000A976DD | 'URS0000A976DD' | 3.860704 | 1.57E-07 |
|  | URS0000A9BCF4 | 'URS0000A9BCF4' | -1.74933 | 1.83E-21 |
|  | URS0000B83B85 | 'URS0000B83B85' | 1.333229 | 6.80E-42 |
|  | URS0000D6F4B8 | 'URS0000D6F4B8' | 6.409447 | 2.32E-10 |
| ncRNA | 100502933 | 'Gm19461' | -1.13588 | 7.53E-15 |
|  | 100504177 | 'F730016J06Rik' | -1.31629 | 3.20E-04 |
|  | 102633008 | 'Gm38450' | 1.235309 | 1.94E-04 |
|  | 102633192 | 'Gm31080' | -1.2613 | 1.15E-06 |
|  | 102633226 | 'Gm31107' | -1.04846 | 3.34E-04 |
|  | 102633433 | 'Gm29478' | -1.26917 | 5.57E-06 |
|  | 102635184 | 'Gm27572' | 2.374681 | 6.29E-06 |
|  | 102635461 | 'Gm32793' | 1.155426 | 3.17E-17 |
|  | 102635607 | 'Gm32898' | -1.32526 | 1.04E-04 |
|  | 102635703 | 'Gm32974' | 1.997408 | 2.76E-10 |
|  | 102636547 | 'Gm33583' | -1.45931 | 6.97E-05 |
|  | 102636754 | 'Gm33738' | -1.06188 | 2.03E-13 |
|  | 102638542 | 'Gm35079' | 5.104592 | 1.37E-04 |
|  | 102638916 | 'Gm35362' | -1.19306 | 2.33E-07 |
|  | 102640498 | 'Gm36543' | 1.727623 | 9.56E-04 |
|  | 102640765 | 'Gm36757' | -1.1986 | 3.54E-04 |
|  | 102640777 | 'Gm36763' | -1.15395 | 2.23E-05 |
|  | 102641105 | 'Gm38534' | -1.2974 | 7.84E-08 |
|  | 105243242 | 'Gm39215' | -1.35727 | 8.40E-05 |
|  | 105243280 | 'Gm39244' | -1.10573 | 3.22E-13 |
|  | 105243330 | 'Gm39283' | -1.02155 | 9.43E-08 |
|  | 108168492 | 'Gm46693' | 1.117977 | 4.55E-04 |
|  | 108168696 | 'Gm46721' | -1.11579 | 3.23E-04 |
|  | 108168820 | 'Gm34294' | -1.1812 | 5.22E-11 |
|  | 108168890 | 'Gm46819' | -2.1559 | 2.13E-05 |
|  | 115487132 | 'LOC115487132' | 1.366279 | 4.12E-07 |
|  | 320671 | 'D130079A08Rik' | -1.07379 | 3.81E-04 |
|  | 75990 | '5033421B08Rik' | -2.19232 | 6.20E-05 |
| C_region | 100125263 | 'Trbc2' | 1.466437 | 1.99E-05 |
